# Genome-wide estimation of recombination, mutation and positive selection enlightens diversification drivers of *Mycobacterium bovis*

**DOI:** 10.1038/s41598-021-98226-y

**Published:** 2021-09-22

**Authors:** Ana C. Reis, Mónica V. Cunha

**Affiliations:** 1 Centre for Ecology, Evolution and Environmental Changes (cE3c), Faculdade de Ciências, Universidade de Lisboa, Campo Grande, C2, Room 2.4.11, 1749-016 Lisbon, Portugal; 2 Biosystems and Integrative Sciences Institute (BioISI), Faculdade de Ciências da Universidade de Lisboa, Lisbon, Portugal

**Keywords:** Computational biology and bioinformatics, Evolution, Microbiology

## Abstract

Genome sequencing has reinvigorated the infectious disease research field, shedding light on disease epidemiology, pathogenesis, host–pathogen interactions and also evolutionary processes exerted upon pathogens. *Mycobacterium tuberculosis* complex (MTBC), enclosing *M. bovis* as one of its animal-adapted members causing tuberculosis (TB) in terrestrial mammals, is a paradigmatic model of bacterial evolution. As other MTBC members, *M. bovis* is postulated as a strictly clonal, slowly evolving pathogen, with apparently no signs of recombination or horizontal gene transfer. In this work, we applied comparative genomics to a whole genome sequence (WGS) dataset composed by 70 M*. bovis* from different lineages (European and African) to gain insights into the evolutionary forces that shape genetic diversification in *M. bovis*. Three distinct approaches were used to estimate signs of recombination. Globally, a small number of recombinant events was identified and confirmed by two independent methods with solid support. Still, recombination reveals a weaker effect on *M. bovis* diversity compared with mutation (overall r/m = 0.037). The differential r/m average values obtained across the clonal complexes of *M. bovis* in our dataset are consistent with the general notion that the extent of recombination may vary widely among lineages assigned to the same taxonomical species. Based on this work, recombination in *M. bovis* cannot be excluded and should thus be a topic of further effort in future comparative genomics studies for which WGS of large datasets from different epidemiological scenarios across the world is crucial. A smaller *M. bovis* dataset (*n* = 42) from a multi-host TB endemic scenario was then subjected to additional analyses, with the identification of more than 1,800 sites wherein at least one strain showed a single nucleotide polymorphism (SNP). The majority (87.1%) was located in coding regions, with the global ratio of non-synonymous upon synonymous alterations (dN/dS) exceeding 1.5, suggesting that positive selection is an important evolutionary force exerted upon *M. bovis*. A higher percentage of SNPs was detected in genes enriched into “lipid metabolism”, “cell wall and cell processes” and “intermediary metabolism and respiration” functional categories, revealing their underlying importance in *M. bovis* biology and evolution. A closer look on genes prone to horizontal gene transfer in the MTBC ancestor and included in the 3R (DNA repair, replication and recombination) system revealed a global average negative value for Taijima’s D neutrality test, suggesting that past selective sweeps and population expansion after a recent bottleneck remain as major evolutionary drivers of the obligatory pathogen *M. bovis* in its struggle with the host.

## Introduction

The *Mycobacterium tuberculosis* complex (MTBC) is one of the most successful taxon of bacterial pathogens and a paradigmatic case in bacterial evolution, revealing a strikingly high nucleotide identity at the genome level (> 99%) among its members^1,2^. The different MTBC ecotypes cause tuberculosis (TB), an infectious granulomatous disease, in a broad group of host species, ranging from micro-mammals to humans^3–5^. Currently, the complex encompasses human [*M. tuberculosis* (*Mtb*), *M. africanum*] and animal-adapted pathogens (*M. bovis*, *M. caprae*, *M. pinnipedii*, *M. microti*, *M. mungi*, *M. orygis, M. suricattae*, “chimpanzee bacillus” and “dassie bacillus”)^5,6^. *M. canettii* (also known as “smooth tubercle bacilli”) has an average nucleotide identity of 98% with the aforementioned mycobacteria and comparative genomic works suggest that *M. canettii* and the rest of MTBC have diverged very recently from a common ancestor^7^. Considering this notion, several authors refer to *M. canettii* as an MTBC member^8^.

The MTBC has been systematically described as a strictly clonal complex, with population structure being apparently dominated by reductions in diversity, bottlenecks, selective sweeps and genetic drifts^9,10^. Assuming the strictly clonal evolution of the complex, polymorphisms such as deletions cannot be restored by recombination^9^. Based on this premise, the successive events of genomic deletions of the regions of difference (RD) and TbD1 (Mtb specific deletion 1 region) have been proposed as molecular markers of MTBC evolution^2,5,11^. Comparative genomics and whole genome sequencing (WGS) works support the division of human-adapted members into nine lineages (*M. tuberculosis* L1 to L4, L7 and L8; and *M. africanum* L5, L6 and L9), with lineages L2 to L4 sharing the deletion of TbD1 region^2,11–13^. Moreover, animal-adapted members have been proposed to share a common ancestor and are defined by clade-specific deletions in the RD7, RD8, RD9 and RD10^2,5,14^.

Events of horizontal gene transfer (HGT) and recombination are assumed to be rare and to have occurred in the ancestors of MTBC, rather than throughout the diverging history of MTBC members^15–17^. Two early reports by Hughes and collaborators (2002) and Gutacker and collaborators (2006) suggested that recombination events might have helped to shape the polymorphisms marking specific *loci* of *M. tuberculosis* strains^18,19^. The apparent absence of recombination in MTBC has been attributed to: (1) loss of mechanistic processes and ability for HGT; (2) rareness of HGT events; and (3) no opportunity for recombination events within MTBC ecological niches^14,17^. More recently, a few Whole Genome Sequencing (WGS) studies applied to MTBC strains^20^ and *M. bovis*^21^ provided evidences of recombination, with the first suggesting that MTBC strains frequently exchange small DNA fragments, but because of the limited nucleotide sequence variation, these events remain unnoticed.

*Mycobacterium bovis* is the MTBC member most frequently recovered from livestock, mainly cattle, although it can also be isolated from free-ranging and fenced wildlife^4,22–24^. *M. bovis* evolved to five main clonal complexes [European 1 (Eu1), European 2 (Eu2), European 3 (Eu3), African 1 (Af1) and African 2 (Af2)], defined based on spoligotyping profile, specific deletions and single nucleotide polymorphisms (SNPs) in specific genes^25–29^. These clonal complexes evidence the diversity structure of *M. bovis* population and association with geographic regions. Furthermore, a recent WGS work by Zimpel and collaborators (2020) devised an *M. bovis* SNP-based phylogeny with over 1900 genomes, which suggested the existence of at least four distinct lineages in the world (named Lb1 to Lb4), that are not entirely concordant with the previous defined clonal complexes, although geographic specificities may also be confirmed^30^. These authors performed phylogenetic and molecular dating divergence analyses but did not investigate recombination^30^.

Previous works employing different molecular techniques such as spoligotyping, MIRU-VNTR (*Mycobacterial Interspersed Repetitive Unit-Variable Number of Tandem Repeat*) and, more recently, SNP typing, revealed a certain level of genetic diversity among *M. bovis* strains^31–35^. The differentiation of genetic variants has become a crucial tool to study disease epidemiology, contributing to gain insights into pathogenesis, virulence and disease transmission. The arrival of WGS methodologies opened the possibility to shed light into the evolutionary drivers exerted upon *M. bovis* genomes during adaptation and persistence to different hosts and epidemiological scenarios.

In this work, we take advantage of a comparative genomic analysis of a diverse *M. bovis* dataset (*n* = 70), including isolates from different clonal complexes to gain insights into the evolutionary processes of *M. bovis,* specifically addressing phylogenetic relationships and recombination events. Complementary to this analysis, the sub-dataset of *M. bovis* isolates (*n* = 42) obtained from a well characterized multi-host TB endemic region in Portugal^31,36^ was further explored to infer the balance between the relative rates of nonsynonymous (dN) to synonymous (dS) nucleotide substitution, and the evolutionary contribution of specific groups of genes referred to in the literature as having been acquired though HGT by the MTBC ancestor^37,38^, as well as genes encoding 3R (DNA repair, replication and recombination) system components^39^. The genes proposed to be acquired through HGT were selected since they may represent ancient polymorphisms, and so it is expected that they might contain a higher fraction of synonymous alterations. The genes included in the 3R system were selected since previous work performed with *M. tuberculosis* strains suggest a general negative/purifying selection acting upon these genes and that they might play an important role in evolution^39^. Another objective of the work was to infer the presence of recombination events. For this purpose, and considering that our dataset from Portugal only had genomes included in European clonal complex 2 and strains without a clonal complex assigned, we decided to include publicly available genomic data to end up with representatives from all clonal complexes and to increase robustness and breadth of results.

## Methodology

### *Mycobacterium bovis* isolates dataset

Forty-two newly sequenced *M. bovis* genomes from an endemic multi-host TB scenario in Portugal (details below), previously characterized from an epidemiological point of view^36^, were at the centre of this work. Considering that the dataset from Portugal only has representatives of European 2 clonal complex and strains without complex assigned, publicly available whole genome sequencing data was added in order to enlarge the dataset with representatives from all *M. bovis* clonal complexes. Therefore, three sources of whole genome sequencing data were used in this work: complete/draft genome assemblies up to a maximum of 10 scaffolds deposited at NCBI (National Center for Biotechnology Information) (*n* = 15 isolates); Illumina fastq files deposited at SRA (Sequence Read Archive) representative of *M. bovis* clonal complex diversity (*n* = 12 isolates)^30^; and 42 newly sequenced genomes from Portugal. *Mycobacterium bovis* BCG (bacillus Calmette-Guérin) was excluded from the NCBI search. *M. bovis* AF2122/97 commonly used as reference genome was included in the dataset. Due to the public unavailability of whole genome sequences from representatives of African 1 clonal complex, and the low numbers of genomes from representative strains of Af2 and Eu1, raw sequencing data available at SRA was used in those cases. The work of Zimpel and collaborators (2020) helped in the identification of genomes from the aforementioned clonal complexes and in the selection process of *M. bovis* to include in the dataset. For Eu3, only one type genome is described (Branger et al., 2020), thus the genome that we included is the solo representative of the Eu3 complex.

Globally, the dataset included 70 M*. bovis* isolated from eight host species, distributed by 12 countries between 1985 and 2016. Thirty-six were assigned as Eu2, seven as Eu1, one as Eu3, three as Af1, four as Af2 and 19 were not attributed to any clonal complex (details below). Detailed information about the *M. bovis* used in this study (including accession numbers) can be found in Table 1 and Supplementary Table 1.

**Table 1:** Characteristics of *Mycobacterium bovis* genomes used in this work.

*M. bovis* ID	Clonal complex^(a)^	Country	Year	Host species	References	Type of sequence
Mb0220	w/o CC	Portugal	2003	Cattle	^40^	Newly sequenced
Mb0261	Eu2	Portugal	2006	Red deer	^40^	Newly sequenced
Mb0601	Eu2	Portugal	2007	Cattle	^40^	Newly sequenced
Mb0769	Eu2	Portugal	2008	Cattle	^40^	Newly sequenced
Mb0783	Eu2	Portugal	2008	Wild boar	^40^	Newly sequenced
Mb0865	Eu2	Portugal	2008	Cattle	^40^	Newly sequenced
Mb0891	Eu2	Portugal	2009	Red deer	^40^	Newly sequenced
Mb0893	Eu2	Portugal	2008	Wild boar	^40^	Newly sequenced
Mb1317	Eu2	Portugal	2010	Cattle	^40^	Newly sequenced
Mb1339	Eu2	Portugal	2010	Cattle	^40^	Newly sequenced
Mb1458	w/o CC	Portugal	2010	Wild boar	^40^	Newly sequenced
Mb1480	w/o CC	Portugal	2010	Cattle	^40^	Newly sequenced
Mb1654	Eu2	Portugal	2011	Cattle	^40^	Newly sequenced
Mb1670	w/o CC	Portugal	2011	Red deer	^40^	Newly sequenced
Mb1711	Eu2	Portugal	2011	Red deer	^40^	Newly sequenced
Mb1712	Eu2	Portugal	2011	Red deer	^40^	Newly sequenced
Mb1714	Eu2	Portugal	2011	Cattle	^40^	Newly sequenced
Mb1744	w/o CC	Portugal	2012	Wild boar	^40^	Newly sequenced
Mb1746	Eu2	Portugal	2012	Red deer	^40^	Newly sequenced
Mb1758	Eu2	Portugal	2012	Cattle	^40^	Newly sequenced
Mb1769	Eu2	Portugal	2012	Wild boar	^40^	Newly sequenced
Mb1785	Eu2	Portugal	2012	Red deer	^40^	Newly sequenced
Mb1789	Eu2	Portugal	2012	Cattle	^40^	Newly sequenced
Mb1841	Eu2	Portugal	2012	Cattle	^40^	Newly sequenced
Mb1870	Eu2	Portugal	2012	Wild boar	^40^	Newly sequenced
Mb1915	Eu2	Portugal	2013	Red deer	^40^	Newly sequenced
Mb1948	w/o CC	Portugal	2013	Red deer	^40^	Newly sequenced
Mb1960	Eu2	Portugal	2013	Red deer	^40^	Newly sequenced
Mb2026	Eu2	Portugal	2013	Cattle	^40^	Newly sequenced
Mb2043	Eu2	Portugal	2013	Red deer	^40^	Newly sequenced
Mb2067	Eu2	Portugal	2013	Wild boar	^40^	Newly sequenced
Mb2206	Eu2	Portugal	2014	Cattle	^40^	Newly sequenced
Mb2235	w/o CC	Portugal	2014	Red deer	^40^	Newly sequenced
Mb2277	w/o CC	Portugal	2014	Red deer	^40^	Newly sequenced
Mb2300	Eu2	Portugal	2014	Wild boar	^40^	Newly sequenced
Mb2310	Eu2	Portugal	2015	Red deer	^40^	Newly sequenced
Mb2313	Eu2	Portugal	2015	Wild boar	^40^	Newly sequenced
Mb2325	Eu2	Portugal	2015	Red deer	^40^	Newly sequenced
Mb2328	Eu2	Portugal	2015	Red deer	^40^	Newly sequenced
Mb2347	w/o CC	Portugal	2015	Wild boar	^40^	Newly sequenced
Mb2395	Eu2	Portugal	2015	Wild boar	^40^	Newly sequenced
Mb2397	Eu2	Portugal	2015	Wild boar	^40^	Newly sequenced
Mb502499	Af1	Ghana	NA	Human	^30,41^	SRA deposited
Mb502526	Af1	Ghana	NA	Human	^30,41^	SRA deposited
Mb1203064	Af1	Ghana	NA	Human	^30,41^	SRA deposited
Mb4117155	Af2	France	NA	Wild boar	^30,42^	SRA deposited
Mb1791710	Af2	Tanzania	NA	Chimpanzee	^30,43^	SRA deposited
Mb1791712	Af2	Tanzania	NA	Chimpanzee	^30,43^	SRA deposited
Mb1792006	Eu1	USA	2006	Cattle	^43^	SRA deposited
Mb1792127	Eu1	USA	2008	Cattle	^43^	SRA deposited
Mb1792361	Eu1	USA	2013	Cattle	^43^	SRA deposited
Mb7240242	Eu1	USA	2016	Cattle	^43^	SRA deposited
Mb7240415	Eu1	USA	2014	Cattle	^43^	SRA deposited
Mb1791984	Eu1	USA	2005	Cattle	^43^	SRA deposited
MBE1	w/o CC	Egypt	2014	Cattle	NA	assemble/draft genomes NCBI
MBE3	w/o CC	Egypt	2014	Cattle	NA	assemble/draft genomes NCBI
MBE4	w/o CC	Egypt	2014	Cattle	NA	assemble/draft genomes NCBI
MBE10	w/o CC	Egypt	2015	Cattle	NA	assemble/draft genomes NCBI
Mb0077	w/o CC	Canada	2006	Elk	NA	assemble/draft genomes NCBI
Mb0565	w/o CC	Canada	2011	Cattle	NA	assemble/draft genomes NCBI
BMR25	w/o CC	Canada	1985	Bison	NA	assemble/draft genomes NCBI
Mb3601	Eu3	France	2014	Cattle	^29^	assemble/draft genomes NCBI
Mb0476	Eu2	Canada	2002	Cattle	NA	assemble/draft genomes NCBI
MbSP38	Eu2	Brazil	2010	Cattle	^44^	assemble/draft genomes NCBI
Mb1595	w/o CC	Korea	2012	Cattle	^45^	assemble/draft genomes NCBI
Mb0030	w/o CC	China	NA	NA	^46^	assemble/draft genomes NCBI
Mb0001	Eu2	Brazil	2015	Tapirus terrestris	NA	assemble/draft genomes NCBI
Mb0003	w/o CC	India	1986	Cattle	NA	assemble/draft genomes NCBI
Mb31150	Af2	Uganda	NA	Chimpanzee	^30,47^	assemble/draft genomes NCBI

Eu1: European 1, Eu2: European 2, Eu3: European 3, Af1: African 1, Af2: African 2, and w/o CC: without clonal complex.

NA: non-available information.

#### Newly sequenced genomes (dataset from Portugal)

Forty-two newly sequenced *M. bovis* whole genomes originating from animal TB hotspots in Portugal and scattering a period of over 12 years were at the centre of this study, as the underlying wildlife-livestock disease system has been monitored regularly^31,36^ (Supplementary Fig. 1). These strains were isolated from cattle (*n* = 14), red deer (*n* = 16) and wild boar (*n* = 12) from 2003 to 2015, according to the ensuing procedure: animal tissue samples were pooled and processed following the protocol guidelines recommended in the OIE Manual for Terrestrial Animals and inoculated onto Stonebrink and Löwenstein-Jensen pyruvate solid media and liquid medium. Cultures were incubated at 37 °C and inspected weekly for growth for a minimum period of 12 weeks. Colonies were directly stored at glycerol solution at -80ºC. The DNA for the WGS procedure was obtained after a single in vitro passage of original archived samples in mycobacteria selective medium (Middlebrook 7H9, BD Diagnostics). For that purpose, frozen culture stocks were re-cultured on Middlebrook 7H9 supplemented with 5% sodium pyruvate and 10% ADS enrichment (50 g albumin, 20 g glucose, 8.5 g sodium chloride in 1 L water) at 37 °C. After four weeks’ growth, the culture medium was renewed, and the cultures were monitored regularly until growth was observed. Cells were harvested by centrifugation, the pellet was resuspended in 500 µL phosphate buffer saline (PBS), heat-killed at 99 °C during 30 min, centrifuged, and the supernatant stored at -20 °C until WGS. All procedures were performed on a level 3 biosecurity facility.

WGS paired-end genomic libraries were prepared with unique indexing of each DNA sample and sequenced using Illumina MiSeq (2 × 250 pb) (40 samples) and HiSeq (2 × 150 pb) (two isolates) technology (Eurofins Genomics, Germany). The genomic DNA was sequenced using the Illumina Genome Analyser with the paired-end module attachment and libraries were constructed with Nextera XT DNA Library Prep Kit from Illumina, according to the manufacturer’s specifications.

#### Clonal complex assignment

Considering the data recovered from SRA (*n* = 12), the clonal complex identification was available as metadata of the corresponding publications^30,41,43^. When considering complete genomes, with the exception of *M. bovis* AF2122/97 and *M. bovis* 3601 that are recognized members of Eu1 and Eu3 clonal complexes, respectively^25,29^, whole genome alignment with *M. tuberculosis* H37Rv (NCBI accession NC_000962.3) was performed using MAFFT (*Multiple alignment program for amino acid or nucleotide sequences*, version 7.458) with parameter–addfragments^48^. Then, the presence of the deletions and/or SNP characteristic of the different clonal complexes was searched.

The newly sequenced *M. bovis* (*n* = 42) and raw reads from draft assembly genomes (*n* = 3) were aligned with reference genome *M. tuberculosis* H37Rv via vSNP pipeline and the presence of the deletions and/or SNP characteristic of the different clonal complexes was searched.

Information from the presence/absence of characteristic deletions and/or SNP and spoligotyping profile were gathered to assign the genomic data to the corresponding clonal complex. For four draft assemblies it was not possible to infer the spoligotyping profile, and so they were included in the “without complex” group.

### Bioinformatics analysis

**Figure 1:**
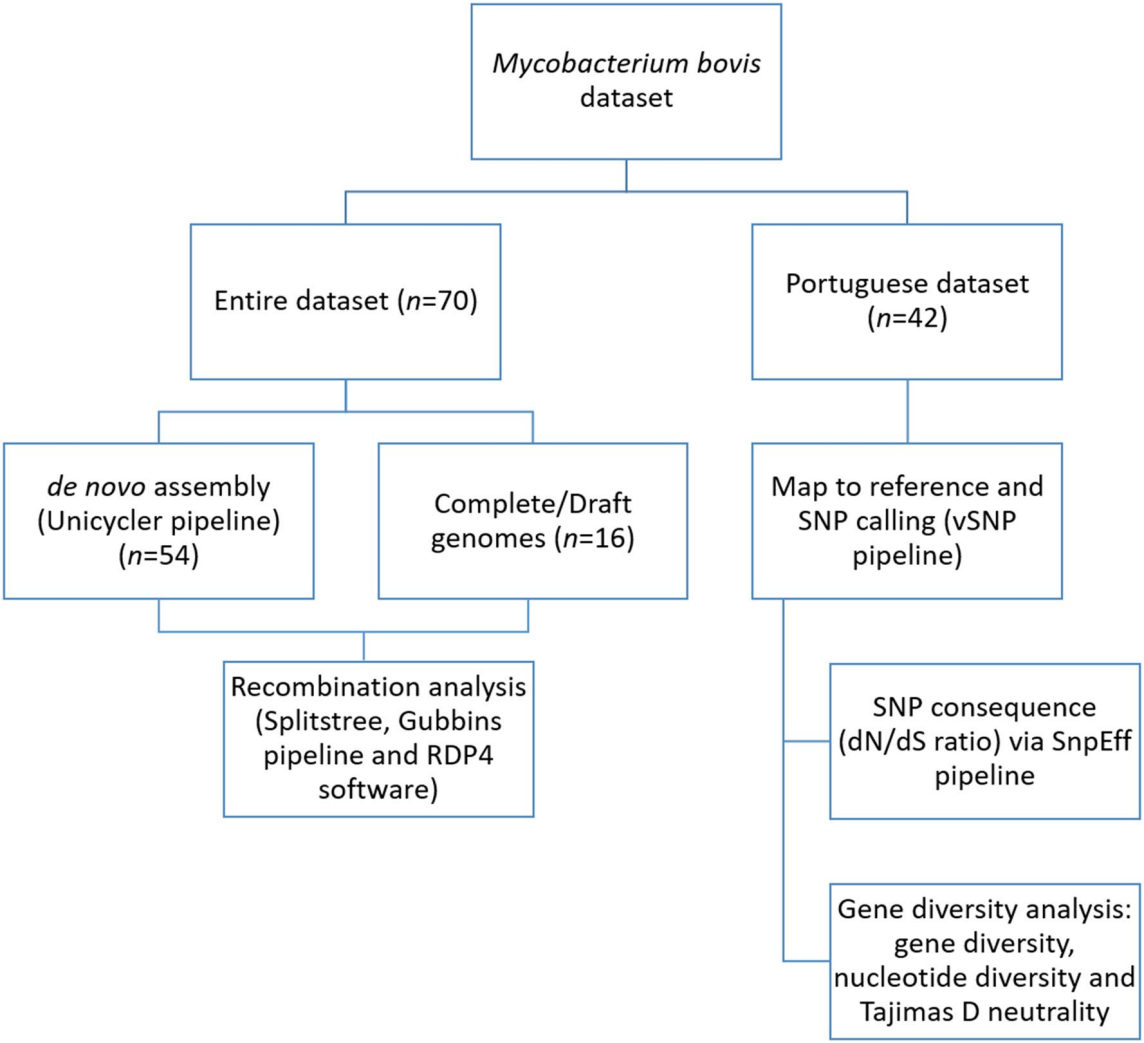
Bioinformatics workflow followed in this study.

The bioinformatics workflow followed in this work started from de novo assembly and map to reference strategies, with the purpose to explore recombination events and the polymorphisms of specific gene groups. Figure 1 provides a flowchart of the steps followed. For the recombination analysis, all the genomes were used to increment the robustness of inferences and the associated metrics.

#### De novo genome assembly

In order to mitigate errors in the generation of genome consensus sequences, we first obtained de novo assemblies and, then, the core multi-alignment. The Unicycler pipeline, currently available at https://github.com/rrwick/Unicycler^49^, was implemented to perform de novo assembly for 54 sequenced genomes (42 newly sequenced and 12 fastq files recovered from SRA). Briefly, before de novo assembly, reads quality analysis was performed in FastQC version 0.11.7 (https://github.com/s-andrews/FastQC), and whenever necessary cleaned with Trimmomatic version 0.36 (options “cut adapter and other illumina-specific sequences from the read” and “cut bases off the end of a read, if bellow a threshold quality of 20” were applied) (http://www.usadellab.org/cms/?page=trimmomatic)^50^. Then, SPAdes optimiser^49^ was used for genome assembly and Pilon version 1.18^51^ for post-assembly optimization. A conservative bridging mode was selected to avoid misassemble and the k-mer size was searched and selected between 20 and 95% of read length. Following SPAdes guidelines and considering reads’ size, contigs with less than 300 bp were removed and a 20 read depth coverage cut-off was established^52^. In the de novo assembly strategy, no genome regions, such as the highly repetitive Proline-Glutamate (PE) and Proline-Proline Glutamate (PPE) paralogous genes, were removed.

The quality of de novo assemblies was assessed by QUAST pipeline (http://quast.sourceforge.net/quast.html), which promotes the remapping of contigs with *M. bovis* AF2122/97 reference genome (NCBI accession number LT708304.1) (quality parameters presented in Supplementary Table 1).

#### Genome map to reference

The FASTQ files from the newly sequenced *M. bovis* obtained from Illumina sequencing were aligned with *M. bovis* AF2122/97 reference genome (LT708304.1) with the help of vSNP pipeline (https://github.com/USDA-VS/vSNP). The standard filtering parameters or variant quality score recalibration were applied according to Genome Analysis Toolkit (GATK)’s Best Practices recommendations^53–55^. Results were filtered using a minimum SAMtools quality score of 150 and AC = 2. Reads were also examined using Kraken (http://ccb.jhu.edu/software/kraken/) to exclude contamination. The vSNP pipeline used for the map to sequence strategy in our work examines a series of defining SNPs and targets also to exclude mixed infection scenarios. Genome coverage by reads was superior to 99% (Supplementary Table 1).

To avoid mapping errors and false SNPs, a variant was filtered out if: (1) it was supported by less than 20 reads, (2) it was found in a frequency of less than 0.9, (3) it was registered in at least one strain but also with a gap in at least another strain. SNPs and positions with mapping issues or alignment problems were visually validated with Integrated Genomics Viewer (IGV) version 2.4.19 (http://software.broadinstitute.org/software/igv/)^56^. Since Proline-Glutamate (PE) and Proline-Proline Glutamate (PPE) genes are highly repetitive and part of multi-gene families, they are prone to misreading by Illumina sequencing and mis-mapping and so are preferentially removed from the bioinformatics workflow of *Mycobacterium tuberculosis* complex members when a strategy of map to sequence is used to confirm SNPs. We thus filtered PE/PPE genes out from the analysis, as well as indels.

All SNPs were grouped into functional categories according with *Bovilist* (http://genolist.pasteur.fr/BoviList/). The SnpEff pipeline (https://pcingola.github.io/SnpEff/) was employed to infer SNP consequences (synonymous or non-synonymous alterations). A new database for *M. bovis* AF2122/97 genome (LT708304.1) was created.

#### Global core genome multi-alignment

The core genome multi-alignment was performed with Parsnp v1.2, currently available at https://github.com/marbl/parsnp^57^, using the 69 complete genomes/draft assemblies (with option -c) and *M. bovis* AF2122/97 (LT708304.1) as reference. Four core multi-alignment were performed: including only members of Eu2 clonal complex (*n* = 37), including all members of European clonal complexes (*n* = 44), including a junction of European and African clonal complexes (*n* = 51), and including all *M. bovis* from this study (*n* = 70).

The core alignments generated by Parsnp were used to infer maximum-likelihood (ML) phylogenetic trees using RAxML, via CIPRES Science Gateway v3.3 (http://www.phylo.org/)^58^, with 1000 bootstrap replications.

#### Estimation of recombination events

The presence of recombination events was examined using three different algorithms and bioinformatics tools in parallel: SplitsTree4 software, Gubbins (Genealogies Unbiased By recomBinations In Nucleotide Sequences) pipeline and RDP4 (Recombination Detection Program, version beta 4.101) software.

The split decomposition method implemented in SplitsTree4 v4.15.1 (http://www.splitstree.org/)^59^ was implemented to compute unrooted phylogenetic networks, which were validated statistically using the Phi test, with a significance threshold of *p* = 0.05. The core multi-alignments from Parsnp analysis were used as input and the split decomposition as network criteria was implemented.

Gubbins pipeline v2.3.1 (https://github.com/sanger-pathogens/gubbins^60^ was run using default parameters, as another way to assess the impact of recombination on *M. bovis*. The algorithm implemented in the pipeline reconstructs the clonal genealogy relating the complete genomes/draft assemblies of our dataset and the reference genome (*M. bovis* AF2122/97, LT708304.1) to each other; and scans the positions of SNPs across each branch of the tree in order to detect clusters of SNPs that would indicate recombination events. The null hypothesis for branch assumes the absence of any recombination events, therefore implying that the SNPs occurring on the branch should be evenly distributed. The core multi-alignments from Parsnp and the best scoring ML tree from RAxML were used as input files.

Finally, to confirm the recombination events suggested by the Gubbins pipeline, six algorithms (RDP^61^, GENECONV^62^, Bootscan^63^, Maxchi^64^, Chimaera^65^, and SiScan^66^) implemented in RDP4^67^ were applied to the core multi-alignments from Parsnp under default settings. We established that at least three of the algorithms implemented in RDP4 had to concordantly evidence a significant signal to validate each recombination event.

Considering that both Gubbins and RDP software seek recombination signals by inspecting the core multi-alignment in windows of 500 bp maximum, and to confirm that the inclusion of PE/PPE genes in the de novo assembly process did not interfere with the recombination signals found, the neighbourhood of genes in which recombination events were identified were further inspected through a synteny analysis. Synteny maps, using complete genomes, were constructed with MAUVE—multi-genome alignment (http://darlinglab.org/mauve/mauve.html) to exclude local genome translocations or inversions. Furthermore, a synteny analysis with aminoacidic sequences was performed via SyntTax webserver (https://archaea.i2bc.paris-saclay.fr/SyntTax/) using complete genomes.

#### Gene diversity analyses

The genome dataset obtained from a multi-host TB system in Portugal was subjected to deeper analyses with the objective to examine the polymorphisms in the genes referred in the literature as having been acquired through HGT by the MTBC ancestor^37,38^ and in the genes encoding 3R (DNA repair, replication and recombination) system components^39^. Gene sequences of the 42 M. *bovis,* together with gene sequence from the reference genome (*M. bovis* AF2122/97, NC_002945.4), were aligned using ClustalX v2.1 (http://www.clustal.org/clustal2/) and used as an input for the calculation of gene diversity, nucleotide diversity (π) and Tajima's D neutrality test parameters via DnaSP v6.12.03 (http://www.ub.edu/dnasp/).

## Results and discussion

### Global phylogenetic analysis

**Figure 2:**
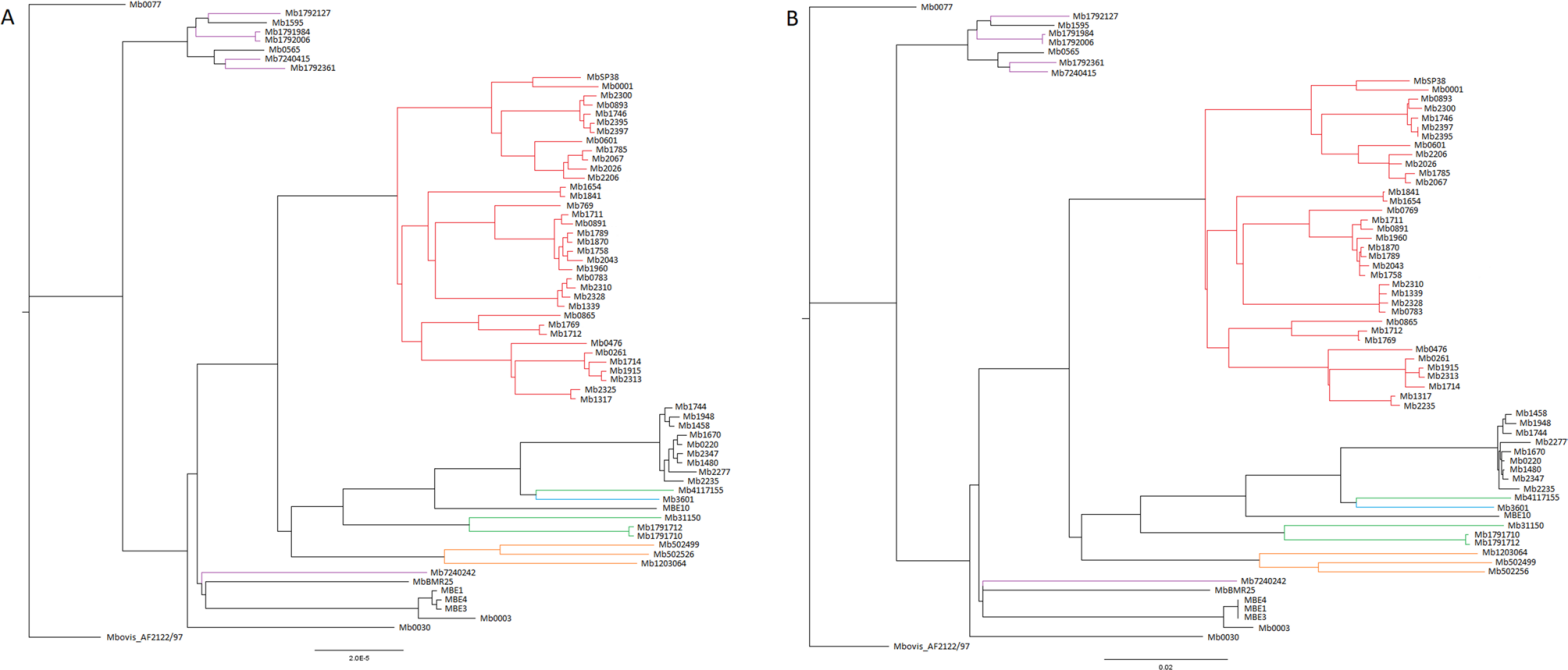
Maximum likelihood phylogenetic tree (GTR) built based on core-genome alignment of *M. bovis* genomes before (**A**) and after (**B**) the removal of recombination sites. Branch colors represent *M. bovis* clonal complexes: purple for European 1, red for European 2, blue for European 3, orange for African 1 and green for African 2. The tree is rooted and drawn to scale with branch lengths measured as the number of substitutions per site.

A Maximum Likelihood (ML) phylogenetic tree based on the 69 M*. bovis* isolates and reference genome was obtained (Fig. 2A). This strategy allows the generation of a more robust tree, when comparing with single gene based trees or multi-locus based trees, that do not capture the variability across the entire genome and consequently present low inter-specific discriminatory power^68,69^. The resulting topology of the ML tree generally agrees with clonal complex classification, with genomes of Eu2 clustering in one tree branch and genomes of Af1 also clustering together (Fig. 2A). Results are also in agreement with the known *M. bovis* evolutionary relationships that present a large division between Eu1 members and a group composed by all the other clonal complexes and genomes without assigned clonal complex^30^. Small inconsistencies between clonal complex and the relationships observed at the phylogenetic tree can be explained by the fact that clonal complexes are described based on specific genomic regions, while the phylogenetic tree is based on core genome multi-alignment representing the whole genomes.

### Evidences of recombination in *Mycobacterium bovis*

*Mycobacterium tuberculosis* complex is described to have clonally evolved, and most evidences accumulated over the years support the idea that ongoing HGT and recombination events do not occur at detectable levels in the MTBC^15,17,18^.

Previous works have suggested that there might be limited recombination among MTBC strains^20,21^, while others were not successful to identify measurable recombination events^70,71^. To revisit this issue with focus on *M. bovis*, and unlike previous works that only accounted for *M. tuberculosis*^70,71^; or that accounted MTBC as a whole, with few *M. bovis* representatives^20^; or that only considered a restrict *M. bovis* dataset^21^, in this work a total of 70 strains, with representatives from all clonal complexes, was used to screen for recombination. The dataset was scaled in four cumulative levels: (1) Eu2 members, (2) all European clonal complexes members (i.e. *European*), (3) both European and African clonal complexes (Eu + Af) and (4) the entire dataset (encompassing the genomes that are not included in any of the clonal complexes already described).

**Figure 3:**
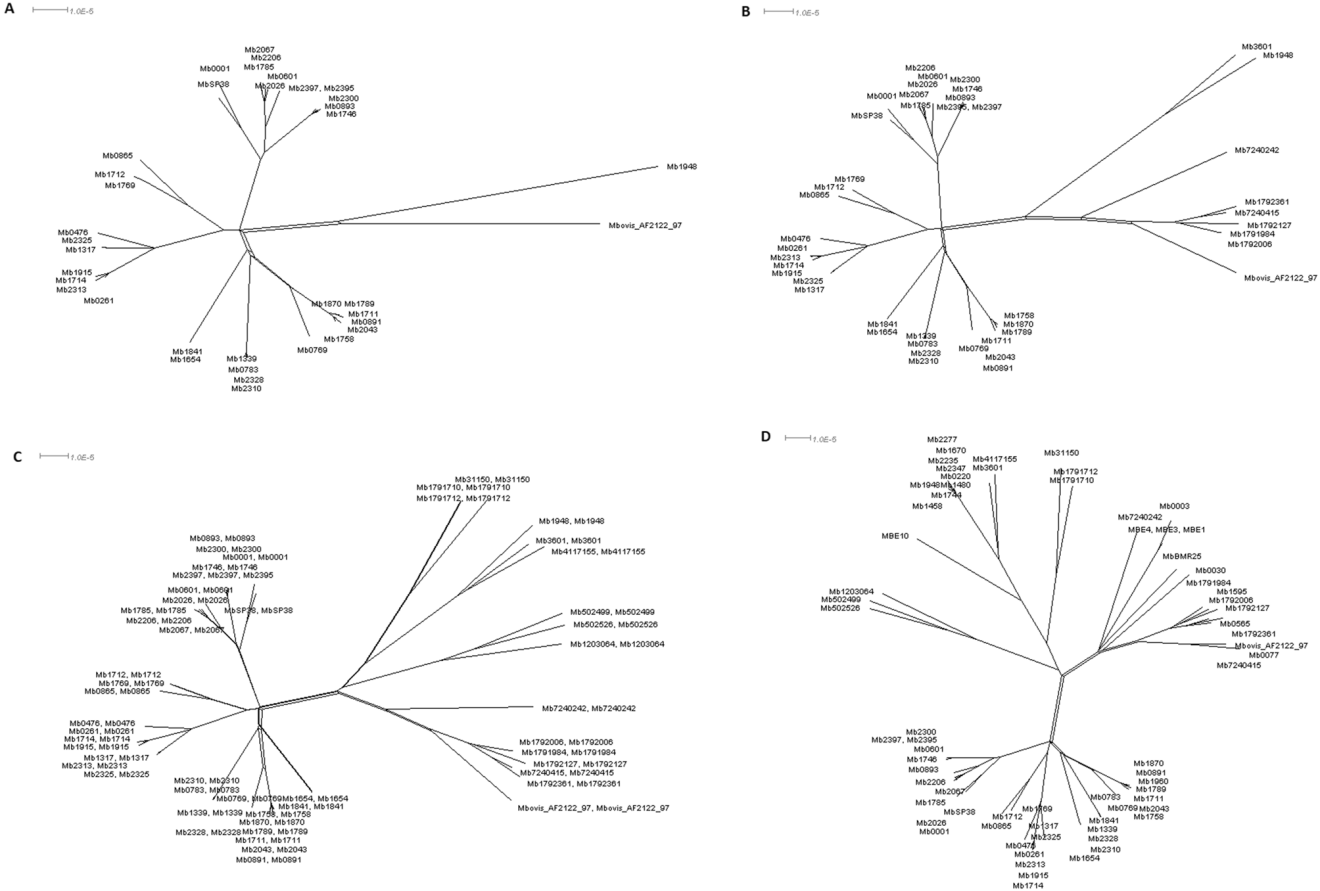
Visualization of conflicting phylogenetic signals at unrooted phylogenetic trees by the split decomposition method in European 2 genomes (*n* = 37) (**A**), in European genomes (*n* = 44) (**B**), in a combination of European and African genomes (*n* = 51) (**C**) and in the entire dataset (*n* = 70) (D).

To investigate this postulate further, a split decomposition network was performed to assess for the absence of recombination events between genomes, since this method enables the visualization of ancestral relationships between individuals and displays conflicting phylogenetic signals. The presence of cycles in the network (i.e. regions that do not converge into a single tree), was confirmed in all four datasets under analysis, however none was supported statistically by the Phi test (Eu2, *p* = 0.0956; European, *p* = 0.1637; Eu + Af *p* = 0.2774; entire dataset *p* = 0.2451), providing poor evidence for the presence of recombination events (Fig. 3A-D).

Following this analysis, and considering the observation of cycles in all networks, the reconstruction algorithm implemented in Gubbins pipeline was applied in order to reconstruct the clonal genealogy and to perform a complementary estimation of the impact of recombination in *M. bovis* genomes. A cumulative number of recombination events was inferred with the majority occurring in terminal branches (i.e. occurring in a single genome) (Table 2). The metrics showed consistency across the datasets and revealed that recombination events occurred two hundred to three hundred times less frequently than mutations, once the rho/theta parameter that represents the relative rates of recombination and point mutation on a branch presented an average value between 0.0037 and 0.0056 (Table 3). Recently, a published work with 38 *M. bovis* strains evidenced a higher rho/theta value (rho/theta = 0.1) than the one obtained for this dataset^21^, however the work by Patané and co-workers used reference-based assemblies to infer recombination parameters, a procedure detail that was already associated with enrichment of putative recombination events at terminal branches due to the assembly procedure^70^.

**Table 2:** Number of recombination events inferred by the Gubbins pipeline and RDP4.

Dataset	No. Gubbins events (% in terminal branches)	No. RDP4 events (% in terminal branches)
European 2 (*n* = 37)	4 (50%)	1 (0%)
European (*n* = 44)	5 (60%)	2 (0%)
European and African (*n* = 51)	6 (66.7%)	2 (0%)
Entire dataset (*n* = 70)	8 (75%)	3 (33.3%)

**Table 3:** Recombination metrics obtained through the Gubbins pipeline analysis.

Dataset	r/m	Rho/theta
European 2 (*n* = 37)	0.025	0.0037
European (*n* = 44)	0.034	0.0046
European and African (*n* = 51)	0.037	0.0056
Entire dataset (*n* = 70)	0.037	0.0044

Following, the r/m parameter, which represents the ratio of diversity introduced by recombination and mutation, revealed an average value between 0.025 and 0.037, pointing that recombination has a lower overall effect in *M. bovis* genetic diversity when comparing with mutation (Table 3). To make a broad comparison, the r/m parameter was estimated using a similar methodology for an MTBC dataset composed by 23 genomes, revealing a mean value of 0.486^20^, while for the 38 M*. bovis* dataset of Patané and co-workers^21^ it evidenced a mean value of 0.98. In the first study there were only two *M. bovis* (*M. bovis* BCG and reference strain) within the 23 genomes included in the work, so the obtained value might be biased by the overrepresentation of *M. tuberculosis* genomes. In the second report, the *M. bovis* population under analysis was mainly recovered from American countries and livestock hosts. In contrast, in our dataset, a higher number of geographic locations and host species is represented, and genomes grouped into different clonal complexes with distinct population genetic signatures were also used, enabling a deeper and wider population knowledge. The differential r/m average values obtained with our dataset are consistent with the notion that the extent of recombination vary widely among lineages assigned to the same taxonomical species, so these results suggest that *M. bovis* clonal complexes might exhibit a differential impact of recombination, as also suggested by Didelot & Maiden^72^. Nevertheless, enlarging significantly this dataset with the inclusion of a higher number of *M. bovis* genomes would allow further clarification of this point. Both r/m and rho/theta parameters present variability among the tree branches, a result that is in agreement with reports concerning other bacterial species^72,73^.

Finally, to confirm the recombination events identified by Gubbins pipeline, the different core multi-alignments were also independently tested in RDP4 software with six different algorithms. Globally, less than half of the events identified by Gubbins were confirmed by RDP4 (Tables 4, 5). Considering the entire dataset, three recombination events were confirmed, two involving internal nodes and another one involving a single genome in a terminal branch and for which a clonal complex could not be assigned (Tables 4, 5). The identification of events in terminal branches might be a sign that recombination is still ongoing in contemporary *M. bovis* strains or the result of misalignment^70^. In this putative recombination region, circa 20% of positions have an undefined nucleotide (N), which can therefore influence the recombination signal (Supplementary Fig. 2). Moreover, this region affects the *rrs* gene, encoding the 16S ribosomal RNA that is expected to be highly conserved, so this putative recombination signal could be the result of a sequencing error or wrong alignment. Whole genome alignment between Mb0003 and *M. bovis* AF2122/97 was thus then performed and the presence of undefined nucleotides and of SNPs was confirmed, so the likely issues related to wrong alignment did not arrive as a consequence of the bioinformatics procedure implemented in this work.

**Table 4:** Detailed information concerning the recombination events identified by Gubbins and RDP4 in the entire dataset.

Recombination event	Identification	Core-alignment positions	Genome positions^(a)^	Gene name	Mb gene name	Classification of gene function	*M. bovis* isolate ID
#1	Gubbins	945,923–945,950	1,220,297–1,220,324	PE PGRS22	Mb1121	PE-PGRS family protein	Mb2026
#2	Gubbins; RDP4	1,176,674–1,177,221	1,475,305–1,475,975	*rrs*	Mb5019	Ribosomal RNA 16S	Mb0003
#3	Gubbins; RDP4	1,532,736–1,532,787	1,953,495–1,953,548	*narX*	Mb1765c	Probable nitrate reductase NarX	Mb1792361 Mb7240415
#4	Gubbins	1,532,751–1,532,781	1,953,840–1,953,870	*narX*	Mb1765c	Probable nitrate reductase NarX	Mb1792361
#5	Gubbins; RDP4	1,794,609–1,794,714	2,283,200–2,283,315	*pks12*	Mb2074c	Probable polyketide synthase pks12	Mb0891 Mb1711 Mb1789 Mb1870 Mb1758 Mb2043 Mb1960
#6	Gubbins	1,794,627–1,794,780	2,283,713–2,285,136	*pks12*	Mb2074c	Probable polyketide synthase pks12	Mb0003
#7	Gubbins	2,242,002–2,242,098	2,839,474–2,839,570	*tatA*	Mb2121	Probable Sec-independent protein translocase membrane-bound protein tatA	Mb0565
#8	Gubbins	3,244,551–3,244,556	4,003,420–4,003,425	*espa*	Mb3646c	Conserved hypothetical alanine and glycine rich protein	Mb2043

Genome positions according with *M. bovis* AF2122/97.

**Table 5:** Statistical values associated with different algorithms implemented in RDP4 for the confirmed recombination events.

Recombination event		Alignment positions		RDP (*p*-value)		GENECONV (*p*-value)		Bootscan (*p*-value)		MaxChi (*p*-value)		Chimaera (*p*-value)
#2		1,176,674–1,177,221		7.524 × 10^−22^		1.871 × 10^−20^		1.004 × 10^−15^		9.926 × 10^−05^		9.753 × 10^−05^
#3		1,532,736–1,532,787		3.771 × 10^−09^		5.216 × 10^−08^		5.634 × 10^−03^		–		–
#5		1,794,609–1,794,714		1.338 × 10^−11^		2.324 × 10^−10^		6.200 × 10^−12^		–		–

**Figure 4:**
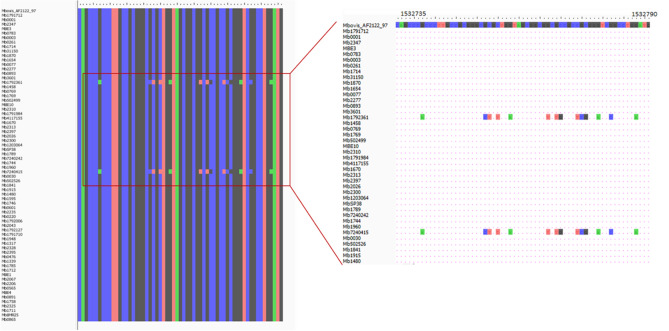
Detailed visualization of alignment in the recombination region of *M. bovis* dataset affecting the *narX* gene encoding a probable nitrate reductase. No gaps or undefined nucleotides were identified in the recombination region of internal nodes. This specific event was registered across Eu1 genomes. The quality of sequencing of *narX* gene was evaluated by read mapping against *M. bovis* AF2122/97. The SNP positions suggested in the recombination region were confirmed by applying the criteria referred to in the methods section (at least 20 reads and 0.9 frequency of alteration). The polymorphisms at *narX* gene were fully confirmed in genomes Mb1792361 and Mb7240415 (2.3%).

**Figure 5:**
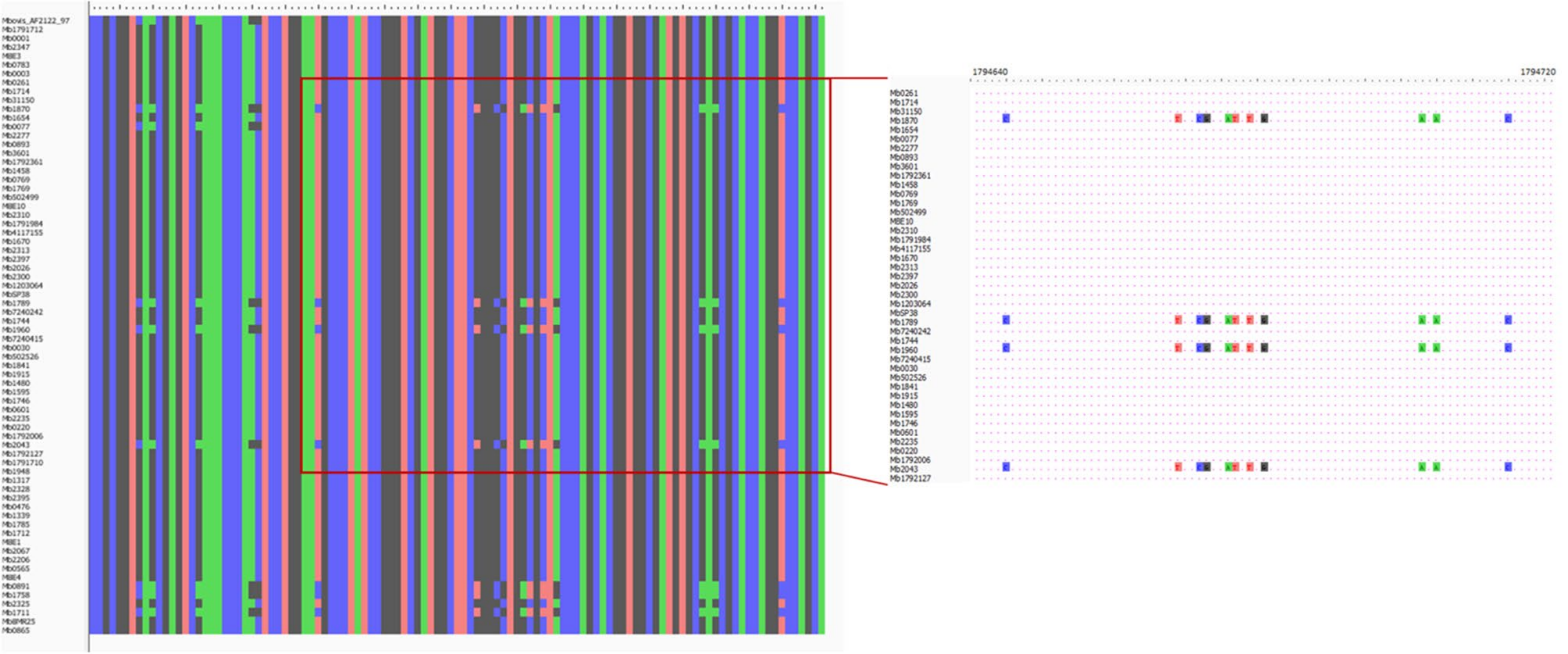
Detailed visualization of alignment in the recombination region of *M. bovis* dataset affecting the *pks12* gene. No gaps or undefined nucleotides were identified in the recombination region of internal nodes. With respect to this event affecting the *pks12* gene that encodes a probable polyketide synthase, it encompasses exclusively Eu2 genomes. The quality of sequencing of *pks12* was evaluated by read mapping against *M. bovis* AF2122/97. The SNP positions suggested in the recombination region were confirmed by applying the criteria referred to in the methods section (at least 20 reads and 0.9 frequency of alteration). The polymorphisms were fully confirmed for genomes Mb0891, Mb1711, Mb1789, Mb1870, Mb1758, Mb2043, Mb1960.

No gaps or undefined nucleotides were identified in the recombination regions of internal nodes (Figs. 4, 5). With respect to these events, one encompasses exclusively Eu2 genomes, affecting the *pks12* gene that encodes a probable polyketide synthase; while the other one is registered across Eu1 genomes and affects *narX* gene encoding a probable nitrate reductase (Table 4). Overall, the recombination analysis suggested the presence of a limited number of recombination segments with statistical support, and the inferred metrics indicate a lower effect of recombination on *M. bovis* genealogy. The recombination signal was expected to be low, however it is important to distinguish true evolutionary signals from background noise, which is a challenging task. In order to decrease the noise signal proposed to be introduced by reference-based assemblies and misalignment issues^70,71^, with the exception of complete genomes, all the remaining ones were de novo assembled and the quality of assemblies was checked and secured via QUAST pipeline analysis (Supplementary Table 1). Moreover, a series of complementary analyses was performed to provide robustness and accurateness to the overall investigation. Thus, the quality of sequencing of *narX* and *pks12* genes was evaluated by read mapping against *M. bovis* AF2122/97. The SNP positions suggested in the recombination region were confirmed by applying the criteria referred in the methods section (at least 20 reads and 0.9 frequency of alteration). The polymorphisms at *narX* gene were fully confirmed in two genomes (Mb1792361 and Mb7240415; 2.3%), as well as in the case of *pks12* gene for genomes Mb0891, Mb1711, Mb1789, Mb1870, Mb1758, Mb2043, Mb1960. However, for genome Mb2043, six out of eight positions did not meet the read depth criteria because the SNPs were supported by a maximum of 17 reads that was below the established cut-off of 20. Recombination at this genome spot could thus be confirmed for six genomes (8.6%) (Figs. 4, 5).

PE and PPE genes have repetitive regions prone to misreading by Illumina sequencing and mis-mapping and so are commonly removed from the bioinformatics workflow of *Mycobacterium tuberculosis* members only when a strategy of map to sequence is used. The inference of recombination events applied in this work was based on de novo assemblies for which PE/PPE were not filtered out. We believe that the strategy applied, with the implementation of three different, complementary approaches and algorithms by SplitsTree, Gubbins pipeline and RDP4 software, is robust to deal and filter recombination regions arising from false signals. Nevertheless, to exclude the interference of PE/PPE genes on the identification of SNP clusters by Gubbins and RDP4 software, and consequently on the identification of the recombination regions proposed to affect *narX* and *pks12* genes, the neighbourhood of these genes was inspected (Supplementary Fig. 3–5). In *M. bovis* AF2122/97, the *narX* gene is delimited by *narK2* and Mb1764c, while *pks12* is surrounded by Mb2075c e Mb2073c (Supplementary Fig. 3–5). Synteny maps with MAUVE using complete genomes yielded plots providing information about gene order conservation and rearrangements, showing four colinear blocks, without signs of genome translocations or inversions. Furthermore, a complementary analysis with aminoacidic sequences evidenced synteny in all complete genomes and no PE/PPE were identified in the neighbourhood regions of *narX* or *pks12*. For *narX*, one genome (Mb0030) had a lower synteny score, since *narX* gene is identified in two segments (segment 1891 and 1890). For *pks12*, Mb0030 and Mb003 present lower synteny scores due to a similar situation, whereas *pks12* is identified in two and three segments, respectively, representing different domains of the protein (Supplementary Fig. 3–5). Considering this information and that both Gubbins and RDP4 software perform an analysis inspecting the core multi-alignment in windows with a maximum of 500 bp, we confirmed that the PE/PPE genes did not interfere with the recombination signals affecting *narX* and *pks12*.

Although the recombination signals detected in this dataset may be considered residual, recombination in *M. bovis* cannot indeed be excluded and should thus continue to be the subject of further analyses for which sequencing of whole genomes from different epidemiological scenarios is crucial.

Comparing the obtained ML phylogenetic trees before and after the recombination correction (Fig. 2A,B) did not lead to significant changes in the inferred phylogenetic relationships, with *M. bovis* strains being gathered within the same groups.

### An evolutionary scenario for *M. bovis* from a multi-host TB system in Portugal

**Figure 6:**
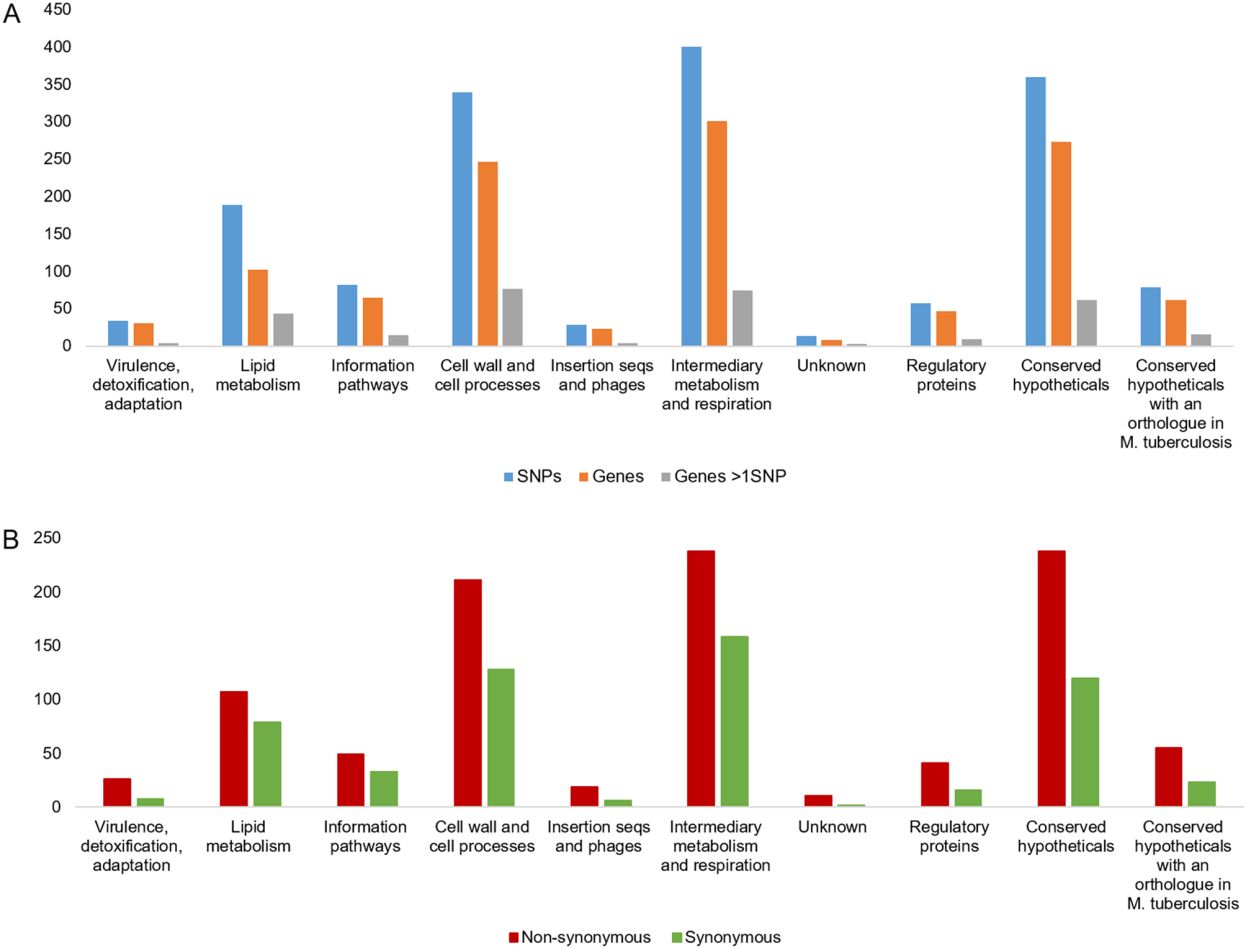
Stratified analysis for the *M. bovis* dataset from Portugal (*n* = 42). Total number of SNPs and affected genes registered *per* functional category (**A**). Total number of synonymous and non-synonymous alterations registered by functional category (**B**).

A SNP alignment containing 1816 polymorphic positions was obtained after mapping reads of 42 newly sequenced *M. bovis* against the reference genome of *M. bovis* AF2122/97. The majority of SNPs (87.1%) was located in coding regions and the affected genes were characterized according to functional categories displayed in *Bovilist* (Fig. 6A,B). After accounting for the total number of genes per functional category, the genes encompassed in “Lipid metabolism” category presented the higher number of SNPs, followed by “Cell wall and cell process” and “Intermediary metabolism and respiration”, revealing their underlying importance in *M. bovis* evolution.

Globally, the average dN/dS ratio is superior to 1.5, which suggests a global evolutionary pressure to escape from the ancestral state and representing positive (diversifying or directional) and/or relaxed purifying selection scenarios. In the categories “Virulence, detoxification, adaptation”, “Insertion seqs and phages” and “Regulatory proteins”, over two-thirds of SNPs were non-synonymous (Fig. 6B).

In all categories, there were genes with more than one SNP, leading to an average rate of mutation (i.e. the mean value of SNPs per gene) greater than one (Fig. 6A). The higher mutation values were harboured by *pks12* (*Mb2074c*) with 15 SNPs and *fas* (*Mb2553c*) with 8 SNPs. Both genes are involved in fatty acid metabolism. The *pks* genes encode polyketide synthases (PKS) which are multifunctional enzymes involved in the biosynthesis of mycobacterial cell wall lipids^74,75^. This gene encodes a multifunctional polypeptide that is involved in the synthesis of mycoketides^74,76^. The *fas* gene is involved in the synthesis of mycolic acids. Both genes play an import role in the biosynthesis of the cell wall that is at the interface with the host.

### SNP-detailed analysis of HGT and 3R genes

To further study the evolutionary processes within *M. bovis,* two specific groups of genes were analysed. Previous published works using sequence composition and phylogenetic methods identified genes that were acquired through HGT by the MTBC ancestor before diversification^37,38^. Those genes are listed in Supplementary Table 2. The SNP distribution was analysed in a total of 77 genes presumably involved in HGT, and 26 polymorphic sites were identified, leading, in the majority of cases (78%), to a non-synonymous (NS) change (Supplementary Table 2). Previous work conducted with MTBC genomes evidenced that putative HGT regions present a higher ratio of NS SNPs when comparing with the rest of the genome^20^. If one considers that these recombination tracts were acquired by the MTBC ancestor and, thus, they over-represent ancient polymorphisms, then it would be expected a higher fraction of synonymous alterations, since NS substitutions are expected to be eliminated by negative selection, as the changes in amino acid might modify protein function. So, our results suggest that functional consequences may arise from substitutions in HGT-like genes, which remits to their importance on valuable adaptive genetic diversity.

In parallel with this analysis, the genes encoding 3R (DNA repair, replication and recombination) system components were thoroughly examined, following the previous published list by dos Vultos and collaborators (2008)^39^. The exchanges of identical DNA fragments cannot be directly observed, although it might be a frequent process when involving closely related bacteria, such as in the case of this dataset; plus, this process might be crucial as a DNA repair method^72^ and thus play a role in homologous recombination. A total of 26 polymorphic positions distributed by 54 genes were identified (Supplementary Table 3). In this group of genes, NS changes account for about 65% of the consequences, which is in agreement with a previous report for *M. tuberculosis* strains^39^.

Gene and nucleotide diversity (π) were evaluated for the genes presenting polymorphisms. Gene diversity is a measure of the uniqueness of a particular gene sequence in a population. Average values of 0.256 and 0.226 were obtained for HGT and 3R group genes, respectively. When the value of gene diversity index is zero, all the sequences under analysis are equal. Therefore, the values obtained in this work reveal that there is limited genetic diversity within the selected panel of genes. The nucleotide diversity (π) compares the similarity per site between two nucleotide sequences. When π is superior to 0.003 it can be considered that the group of sequences under analysis is highly diverse. In our analysis, both gene groups reveal an average value inferior to 0.003, with HGT registering 0.00034 and the 3R *circa.* 0.00021. No gene had a π value higher than 0.003, thus also confirming limited nucleotide diversity within the selected gene panels.

The Tajima's D test of neutrality was also evaluated, and in both groups there were genes with positive and negative values, evidenced by an average value inferior to zero. The selection against deleterious mutations, past selective sweeps and population expansion after a recent bottleneck are pointed as possible causes to decrease the result from Tajima’s D test.

### Balance of forces in *M. bovis* evolution

Natural selection is a mechanism of evolution and has been associated with MTBC evolution^9^. Selective sweeps (i.e. positive selection that leads to the fixation of a new beneficial mutation) and background selection (i.e. selection against a deleterious mutation that leads to the elimination of any mutation linked to the target of selection) are both linked to the action of natural selection.

In this work, several evidences support the importance of natural selection: (1) SNP distribution is not random, with genes included in the “lipid metabolism”, “cell wall and cell processes” and “intermediary metabolism and respiration” categories presenting a higher SNP rate; (2) regions proposed to be transferred from MTBC ancestor also accumulate an excess of SNPs; and (3) the HGT and 3R groups evidenced a global average value inferior to zero in the neutrality tests, indicating a past selective sweep or expansion after bottleneck. Furthermore, the high proportion of low-frequency genetic variants, particularly singletons, is one of the features associated with MTBC population genetics, and proposed to reflect the influence of background selection^10,77^, an effect that is also confirmed in this work, as 372 (20.5%) of the 1816 considered SNPs are strain-specific.

The global elevated value of dN/dS ratio is commonly associated with a positive selection force, likely due to diversifying selection and local selective sweep. However, a reduction in effective population size might have contributed, partially, to this unusual rate of NS per synonymous mutations, once mutations that might have been deleterious in a population with a large effective population size can drift to a high frequency in a small population and, in that way, reflecting reduction in the efficacy of purifying selection as a consequence of increased genetic drift^9,10^.

The affected genes could confer important adaptive advantages through NS substitutions, however functional studies would be necessary to understand the consequences arising from those SNPs and to infer what would be the benefits for mycobacteria. Recent work performed by Yang and collaborators^78^ with *M. tuberculosis* strains suggested that this evolutionary pressure could allow accessory genes (i.e. genes that are not present in all strains or strain-specific genes) to gradually dominate and eventually become core genes (i.e. present in all strains)^79^. This could provide important adaptive and resistance capacities, if considering that accessory genes might be involved in virulence, immune system evasion or antibiotic resistance.

Therefore, a deeper understanding of the role of these evolutionary forces is required to determine which genes have contributed significantly to *M. bovis* evolution in its trajectory of interaction with different hosts in specific disease systems.

## Final conclusions and future work

The study of genetic relatedness and structure of obligatory pathogen populations might provide important insights into their intraspecific genomic diversity and evolution arising upon the interaction with the host. In recent years, many technological advances have shed light onto the biology of *M. bovis*, however the use of high-throughput technologies such as WGS to understand evolutionary steps is still infrequent, with most works in the TB field being focused on *M. tuberculosis* or in the molecular epidemiology of *M. bovis*.

In the current work, a diverse *M. bovis* dataset, with representatives of all described clonal complexes, was used to assess how different evolutionary forces impact and shape the genetic diversity of a population. Altogether, we ended up with a dataset composed of 70 M*. bovis* strains, representing the most diverse dataset available to infer recombination, when comparing with other publicly available works. Furthermore, we used isolates obtained from multiple hosts, including humans. Although we may speculate that the inclusion of more genomes might have an impact on the identification of recombination events and recombination metrics, this pilot work is already significant in the context of present knowledge. More complete analyses may be conducted in the future with larger *M. bovis* datasets to confirm our findings.

The impact of recombination in our dataset was assessed through three complementary strategies. Moreover, efforts to avoid unreliable alignments and to guarantee data quality were made, so that the assessment of recombination signals would be as accurate as possible. Although residual, two approaches support a number of recombination events in the examined dataset, which argue against the paradigm that MTBC is strictly clonal. Despite the limited effects on *M. bovis* diversity when comparing with mutation, recombination events need to be considered in future evolutionary research works in order to further understand their true impact on biological processes, once they may be an important force generating diversification that may translate into virulence, immune evasion and/or antibiotic resistance phenotypes.

Indeed, previous WGS works support recombination in *M. canettii*^7^, showing that strains are highly recombinogenic and evolutionary early-branching, with larger genome sizes, 25-fold more SNPs relative to MTBC members. Those works also provide experimental evidence of how *pks5*-recombination-mediated bacterial surface remodelling in *M. canettii* increased virulence, driving evolution from smooth to rough morphology and from generalist mycobacteria (*M. canetti*) towards professional pathogens of mammalian hosts (MTBC)^80^. Moreover, a recent work performed by Chiner-Oms and collaborators (2019) found evidences of recombination between the MTBC ancestor and *M. canetti* ancestor (before diverging to *M. canettii*), thus proposing the existence of recombination potential before the diversification of MTBC into different ecotypes^71^. So, efforts to expand this topic across all MTBC ecotypes should continue in the future. In this work, we excluded recombination in genomes from the African clonal complexes, nevertheless, a broader sample dataset would be necessary to accurately address the differences amongst clonal complexes members.

Following, the comparative genomic analyses performed in a smaller group of genomes representative of the *M. bovis* population from an endemic TB scenario in Portugal suggested that genes included in the “lipid metabolism”, “cell wall and cell processes” and “intermediary metabolism and respiration” categories have a superior importance in *M. bovis* evolution and a global positive selection force was suggested to be acting upon this population, as informed by the elevated dN/dS ratio^9,10^.

Finally, this work reinforces the value of WGS as a high-resolution tool for the analysis of *M. bovis* genomic diversity and provides insights into the role of recombination and positive selection as evolutionary driving forces in a pathogen affecting a large range of host species, with economical and biodiversity impacts across the world.

## Supplementary Information

Supplementary Information.

## Data Availability

The newly sequencing data included in this work is deposited under the following Biosample accession numbers: SAMN17004141-SAMN17004143, SAMN17004145- SAMN17004174, SAMN17004176- SAMN17004184 and under the Bioproject accession number PRJNA682618 at a public domain server in National Centre for Biotechnology Information (NCBI) SRA database.

## References

[1] Brosch R (2000). Comparative genomics of the mycobacteria. Int. J. Med. Microbiol..

[2] Brosch R (2002). A new evolutionary scenario for the* Mycobacterium tuberculosis* complex. Proc. Natl. Acad. Sci. U. S. A..

[3] Reis AC, Ramos B, Pereira AC, Cunha MV (2020). Global trends of epidemiological research in livestock tuberculosis for the last four decades. Transbound. Emerg. Dis..

[4] Reis AC, Ramos B, Pereira AC, Cunha MV (2020). The hard numbers of tuberculosis epidemiology in wildlife: A meta-regression and systematic review. Transbound. Emerg. Dis..

[5] Brites D (2018). A new phylogenetic framework for the animal-adapted mycobacterium tuberculosis complex. Front. Microbiol..

[6] Gagneux S (2018). Ecology and evolution of *Mycobacterium tuberculosis*. Nat. Rev. Microbiol..

[7] Supply P (2013). Genome analysis of smooth tubercle bacilli provides insights into ancestry and pathoadaptation of the etiologic agent of tuberculosis. Nat. Genet..

[8] Brites D, Gagneux S, Gagneux S (2017). The nature and evolution of genomic diversity in the *Mycobacterium tuberculosis* complex. Strain Variation in the* Mycobacterium tuberculosis* Complex: Its Role in Biology, Epidemiology and Control, Advances in Experimental Medicine and Biology.

[9] Smith NH, Gordon SV, de la Rua-Domenech R, Clifton-Hadley RS, Hewinson RG (2006). Bottlenecks and broomsticks: The molecular evolution of* Mycobacterium bovis*. Nat. Rev. Microbiol..

[10] Hershberg R (2008). High functional diversity in *Mycobacterium tuberculosis* driven by genetic drift and human demography. PLoS Biol..

[11] Bottai D (2020). TbD1 deletion as a driver of the evolutionary success of modern epidemic *Mycobacterium tuberculosis* lineages. Nat. Commun..

[12] Coscolla M (2021). Phylogenomics of *Mycobacterium africanum* reveals a new lineage and a complex evolutionary history. Microb. Genomics.

[13] Ngabonziza JCS (2020). A sister lineage of the *Mycobacterium tuberculosis* complex discovered in the African Great Lakes region. Nat. Commun..

[14] Smith NH (2006). Ecotypes of the *Mycobacterium tuberculosis* complex. J. Theor. Biol..

[15] Liu X, Gutacker MM, Musser JM, Fu YX (2006). Evidence for recombination in *Mycobacterium tuberculosis*. J. Bacteriol..

[16] Rosas-Magallanes V (2006). Horizontal transfer of a virulence operon to the ancestor of *Mycobacterium tuberculosis*. Mol. Biol. Evol..

[17] Gutierrez MC (2005). Ancient origin and gene mosaicism of the progenitor of *Mycobacterium tuberculosis*. PLoS Pathog..

[18] Hughes AL, Friedman R, Murray M (2002). Genomewide pattern of synonymous nucleotide substitution in two complete genomes of *Mycobacterium tuberculosis*. Emerg. Infect. Dis..

[19] Gutacker MM (2006). Single-nucleotide polymorphism-based population genetic analysis of *Mycobacterium tuberculosis* strains from 4 geographic sites. J. Infect. Dis..

[20] Namouchi A, Didelot X, Schöck U, Gicquel B, Rocha E (2012). After the bottleneck: Genome-wide diversification of the *Mycobacterium tuberculosis* complex by mutation, recombination, and natural selection. Genome Res..

[21] Patané JSL (2017). Patterns and processes of *Mycobacterium bovis* evolution revealed by phylogenomic analyses. Genome Biol. Evol..

[22] Naranjo V, Gortázar C, Vicentea J, de la Fuente J (2008). Evidence of the role of European wild boar as a reservoir of *Mycobacterium tuberculosis* complex. Vet. Microbiol..

[23] Palmer MV, Thacker TC, Waters WR, Gortázar C, Corner LAL (2012). *Mycobacterium bovis*: A model pathogen at the interface of livestock, wildlife, and humans. Vet. Med. Int..

[24] Corner LAL (2006). The role of wild animal populations in the epidemiology of tuberculosis in domestic animals: How to assess the risk. Vet. Microbiol..

[25] Smith NH (2011). European 1: A globally important clonal complex of *Mycobacterium bovis*. Infect. Genet. Evol..

[26] Rodriguez-Campos S (2012). European 2—A clonal complex of *Mycobacterium bovis* dominant in the Iberian Peninsula. Infect. Genet. Evol..

[27] Berg S (2011). African 2, a clonal complex of *Mycobacterium bovis* epidemiologically important in East Africa. J. Bacteriol..

[28] Muller B (2009). African 1, an epidemiologically important clonal complex of *Mycobacterium bovis* Dominant in Mali, Nigeria, Cameroon, and Chad. J. Bacteriol..

[29] Branger M (2020). The complete genome sequence of *Mycobacterium bovis* Mb3601, a SB0120 spoligotype strain representative of a new clonal group. Infect. Genet. Evol..

[30] Zimpel CK (2020). Global distribution and evolution of *Mycobacterium bovis* lineages. Front. Microbiol..

[31] Reis AC, Tenreiro R, Albuquerque T, Botelho A, Cunha MV (2020). Long-term molecular surveillance provides clues on a cattle origin for *Mycobacterium bovis* in Portugal. Sci. Rep..

[32] Duarte EL, Domingos M, Amado A, Cunha MV, Botelho A (2010). MIRU-VNTR typing adds discriminatory value to groups of *Mycobacterium bovis* and Mycobacterium caprae strains defined by spoligotyping. Vet. Microbiol..

[33] Hauer A (2015). Genetic evolution of mycobacterium bovis causing tuberculosis in livestock and wildlife in France since 1978. PLoS One.

[34] Conceição EC (2017). Genetic diversity of *Mycobacterium tuberculosis* from Pará, Brazil, reveals a higher frequency of ancestral strains than previously reported in South America. Infect. Genet. Evol..

[35] Chihota VN (2018). Geospatial distribution of *Mycobacterium tuberculosis* genotypes in Africa. PLoS ONE.

[36] Reis AC (2021). Whole genome sequencing refines knowledge on the population structure of *Mycobacterium bovis* from a multi-host tuberculosis system. Microorganisms..

[37] Becq J (2007). Contribution of horizontally acquired genomic islands to the evolution of the Tubercle Bacilli. Mol. Biol. Evol..

[38] Veyrier F, Pletzer D, Turenne C, Behr MA (2009). Phylogentic detection of horizontal gene transfer during the step-wise genesis of Mycobacterium tuberculosis. BMC Evol. Biol..

[39] dos Vultos T (2008). Evolution and diversity of clonal bacteria: The paradigm of *Mycobacterium tuberculosis*. PLoS Negl. Trop. Dis..

[40] Reis, A. C. *et al.* Phylogenomics Sheds Light on the population structure of *Mycobacterium bovis* from a multi-host tuberculosis system. *bioRxiv* 04.26.441523 (2021). 10.1101/2021.04.26.441523 10.3390/microorganisms9081585 PMC8401292 34442664

[41] Otchere ID (2019). Molecular epidemiology and whole genome sequencing analysis of clinical *Mycobacterium bovis* from Ghana. PLoS One.

[42] Branger M (2016). Draft genome sequence of *Mycobacterium bovis* strain D-10-02315 isolated from wild boar. Genome Announc..

[43] Orloski K, Robbe-Austerman S, Stuber T, Hench B, Schoenbaum M (2018). Whole genome sequencing of *Mycobacterium bovis* isolated from livestock in the United States, 1989–2018. Front. Vet. Sci..

[44] Guimarães, A. M. S. *et al.* Draft genome sequence of *Mycobacterium bovis* strain SP38, a pathogenic bacterium isolated from a bovine in Brazil. *Genome Announc.* **3** (2015). 10.1128/genomeA.00511-15 PMC4440967 25999553

[45] Kim N (2015). Complete genome sequence of *Mycobacterium bovis* clinical strain 1595, isolated from the laryngopharyngeal lymph node of South Korean cattle. Genome Announc..

[46] Zhu L (2016). Precision methylome characterization of *Mycobacterium tuberculosis* complex (MTBC) using PacBio single-molecule real-time (SMRT) technology. Nucleic Acids Res..

[47] Wanzala SI (2015). Draft genome sequences of *Mycobacterium bovis* BZ 31150 and *Mycobacterium bovis* B2 7505, pathogenic bacteria isolated from archived captive animal bronchial washes and human sputum samples in Uganda. Genome Announc.

[48] Katoh K, Asimenos G, Toh H (2009). Multiple alignment of DNA sequences with MAFFT. Methods Mol. Biol..

[49] Wick RR, Judd LM, Gorrie CL, Holt KE (2017). Unicycler: Resolving bacterial genome assemblies from short and long sequencing reads. PLoS Comput. Biol..

[50] Bolger AM, Lohse M, Usadel B (2014). Trimmomatic: A flexible trimmer for Illumina sequence data. Bioinformatics.

[51] Walker B (2014). Pilon: An integrated tool for comprehensive microbial variant detection and genome assembly improvement. PLoS One.

[52] Bankevich A (2012). SPAdes: A new genome assembly algorithm and its applications to single-cell sequencing. J. Comput. Biol..

[53] Mckenna A (2010). The genome analysis toolkit : A MapReduce framework for analyzing next-generation DNA sequencing data sequencing data. Genome Res..

[54] Depristo M (2011). A framework for variation discovery and genotyping using next-generation DNA sequencing data. Nat. Genet..

[55] Van der Auwera G (2014). From FastQ data to high confidence variant calls: The Genome Analysis Toolkit best practices pipeline. Curr. Protoc. Bioinforma..

[56] Thorvaldsdóttir H, Robinson JT, Mesirov JP (2012). Integrative Genomics Viewer (IGV): High-performance genomics data visualization and exploration. Brief. Bioinform..

[57] Treangen TJ, Ondov BD, Koren S, Phillippy AM (2014). The Harvest suite for rapid core-genome alignment and visualization of thousands of intraspecific microbial genomes. Genome Biol..

[58] Miller, M. A., Pfeiffer, W. & Schwartz, T. Creating the CIPRES science gateway for inference of large phylogenetic trees. In *Conference paper* (2010). 10.1109/GCE.2010.5676129

[59] Huson DH, Bryant D (2006). Application of phylogenetic networks in evolutionary studies. Mol. Biol. Evol..

[60] Croucher NJ (2015). Rapid phylogenetic analysis of large samples of recombinant bacterial whole genome sequences using Gubbins. Nucleic Acids Res..

[61] Martin D, Rybicki E (2000). RDP: Detection of recombination amongst aligned sequences. Bioinformatics.

[62] Padidam M, Sawyer S, Fauquet CM (1999). Possible emergence of new geminiviruses by frequent recombination. Virology.

[63] Martin DP, Posada D, Crandall KA, Williamson C (2005). A modified bootscan algorithm for automated identification of recombinant sequences and recombination breakpoints. AIDS Res. Hum. Retroviruses.

[64] Smith JM (1992). Analyzing the mosaic structure of genes. J. Mol. Evol..

[65] Posada D, Crandall KA (2001). Evaluation of methods for detecting recombination from DNA sequences: Computer simulations. Proc. Natl. Acad. Sci. U. S. A..

[66] Gibbs MJ, Armstrong JS, Gibbs AJ (2000). Sister-scanning: A Monte Carlo procedure for assessing signals in recombinant sequences. Bioinformatics.

[67] Martin DP, Murrell B, Golden M, Khoosal A, Muhire B (2015). RDP4: Detection and analysis of recombination patterns in virus genomes. Virus Evol..

[68] Devulder G, de Montclos MP, Flandrois JP (2005). A multigene approach to phylogenetic analysis using the genus Mycobacterium as a model. Int. J. Syst. Evol. Microbiol..

[69] Mestre O (2011). Phylogeny of *Mycobacterium tuberculosis* Beijing strains constructed from polymorphisms in genes involved in DNA replication. Recombination and Repair. PLoS One.

[70] Godfroid M, Dagan T, Kupczok A (2018). Recombination signal in *Mycobacterium tuberculosis* stems from reference-guided assemblies and alignment artefacts. Genome Biol. Evol..

[71] Chiner-Oms (2019). Genomic determinants of speciation and spread of the Mycobacterium tuberculosis complex. Sci. Adv..

[72] Didelot X, Maiden MCJ (2010). Impact of recombination on bacterial evolution. Trends Microbiol..

[73] Hadfield J (2017). Comprehensive global genome dynamics of *Chlamydia trachomatis* show ancient diversification followed by contemporary mixing and recent lineage expansion. Genome Res..

[74] Matsunaga I (2004). *Mycobacterium tuberculosis* pks12 produces a novel polyketide presented by CD1c to T cells. J. Exp. Med..

[75] Rousseau C (2003). Virulence attenuation of two Mas-like polyketide synthase mutants of *Mycobacterium tuberculosis*. Microbiology.

[76] Matsunaga I, Sugita M (2012). Mycoketide: A CD1c-presented antigen with important implications in mycobacterial infection. Clin. Dev. Immunol..

[77] Pepperell C (2010). Bacterial genetic signatures of human social phenomena among *M. tuberculosis* from an aboriginal Canadian population. Mol. Biol. Evol..

[78] Yang T (2018). Pan-genomic study of *Mycobacterium tuberculosis* reflecting the primary/ secondary genes, generality/ individuality, and the interconversion through copy number variations. Front. Microbiol..

[79] Vernikos G, Medini D, Riley DR, Tettelin HT (2015). years of pan-genome analyses. Curr. Opin. Microbiol..

[80] Boritsch EC (2016). pks5-recombination-mediated surface remodelling in *Mycobacterium tuberculosis* emergence. Nat. Microbiol..

