# Supplementary material for: Genome-wide estimation of recombination, mutation and positive selection enlightens diversification drivers of Mycobacterium bovis
Source: Sci Rep. 2021 Sep 22;11:18789. doi: 10.1038/s41598-021-98226-y (PMC8458382; doi:10.1038/s41598-021-98226-y)
Supplement: Supplementary file 1 — Supplementary Information. [file 41598_2021_98226_MOESM1_ESM.docx]

**The diversification drivers of *Mycobacterium bovis*: genome-wide estimation of recombination, mutation and positive selection challenge evolutionary paradigms**

Ana C. Reis^1,2^, Mónica V. Cunha^1,2*^

^1^Centre for Ecology, Evolution and Environmental Changes (cE3c), Faculdade de Ciências da Universidade de Lisboa, Lisboa, Portugal

^2^Biosystems & Integrative Sciences Institute (BioISI), Faculdade de Ciências da Universidade de Lisboa, Lisboa, Portugal

*Correspondence: mscunha@fc.ul.pt; Centre for Ecology, Evolution and Environmental Changes (cE3c), Faculdade de Ciências, Universidade de Lisboa, Campo Grande, C2, Room 2.4.11, 1749-016 Lisboa. Phone +351 217 500 000

**Supplementary Table 1.** General information on the *M. bovis* used in this work.

| ***M.* *bovis* ID** | **Bio**  **project** | **Bio**  **sample** | **SRA** | **RefSeq acession code** | **Country** | **Year** | **HS ^(a)^** | **CC ^(b)^** | **No reads** | **No contigs ^(c)^** | **N50 size (bp)^(d)^** | **N75 size (bp)^(d)^** | **NGA50 size (bp)^(d)^** | **NGA75 size (bp)^(d)^** | **Assembly size (bp) ^(c)^** |
| --- | --- | --- | --- | --- | --- | --- | --- | --- | --- | --- | --- | --- | --- | --- | --- |
| Mb0220 | PRJNA682618 | SAMN17004141 | SRR13199762 | NA | Portugal | 2003 | C | w/o CC | 2394434 | 120 | 88279 | 444222 | 81044 | 39419 | 4217943 |
| Mb0261 | PRJNA682618 | SAMN17004142 | SRR13199761 | NA | Portugal | 2006 | RD | Eu2 | 13725892 | 67 | 192315 | 115124 | 192315 | 107061 | 4315875 |
| Mb0601 | PRJNA682618 | SAMN17004143 | SRR13199750 | NA | Portugal | 2007 | C | Eu2 | 1918350 | 115 | 83835 | 42832 | 78286 | 39438 | 4258073 |
| Mb0769 | PRJNA682618 | SAMN17004145 | SRR13199728 | NA | Portugal | 2008 | C | Eu2 | 2047981 | 118 | 81894 | 48289 | 78348 | 42657 | 4265442 |
| Mb0783 | PRJNA682618 | SAMN17004146 | SRR13199723 | NA | Portugal | 2008 | WB | Eu2 | 2012194 | 120 | 84060 | 42405 | 79019 | 39339 | 4257273 |
| Mb0865 | PRJNA682618 | SAMN17004147 | SRR13199722 | NA | Portugal | 2008 | C | Eu2 | 1410917 | 122 | 78990 | 42662 | 65013 | 33833 | 4219925 |
| Mb0891 | PRJNA682618 | SAMN17004148 | SRR13199721 | NA | Portugal | 2009 | RD | Eu2 | 13142182 | 69 | 184570 | 109115 | 184398 | 103753 | 4318176 |
| Mb0893 | PRJNA682618 | SAMN17004149 | SRR13199720 | NA | Portugal | 2008 | WB | Eu2 | 1884560 | 117 | 81563 | 46413 | 78335 | 37615 | 4232720 |
| Mb1317 | PRJNA682618 | SAMN17004150 | SRR13199719 | NA | Portugal | 2010 | C | Eu2 | 1707221 | 115 | 83542 | 48464 | 81342 | 42353 | 4264019 |
| Mb1339 | PRJNA682618 | SAMN17004151 | SRR13199760 | NA | Portugal | 2010 | C | Eu2 | 1640461 | 112 | 91501 | 49547 | 80982 | 43618 | 4265739 |
| Mb1458 | PRJNA682618 | SAMN17004152 | SRR13199759 | NA | Portugal | 2010 | WB | w/o CC | 1929920 | 123 | 73696 | 40242 | 70739 | 37123 | 4268189 |
| Mb1480 | PRJNA682618 | SAMN17004153 | SRR13199758 | NA | Portugal | 2010 | C | w/o CC | 2053130 | 108 | 75870 | 48057 | 70443 | 37281 | 4198146 |
| Mb1654 | PRJNA682618 | SAMN17004154 | SRR13199757 | NA | Portugal | 2011 | C | Eu2 | 615194 | 181 | 52223 | 26632 | 42496 | 23348 | 4192778 |
| Mb1670 | PRJNA682618 | SAMN17004155 | SRR13199756 | NA | Portugal | 2011 | RD | w/o CC | 2074834 | 122 | 69369 | 43081 | 63992 | 39385 | 4262473 |
| Mb1711 | PRJNA682618 | SAMN17004156 | SRR13199755 | NA | Portugal | 2011 | RD | Eu2 | 1758290 | 109 | 83552 | 48330 | 80975 | 42514 | 4251694 |
| Mb1712 | PRJNA682618 | SAMN17004157 | SRR13199754 | NA | Portugal | 2011 | RD | Eu2 | 1085013 | 142 | 63368 | 34683 | 58547 | 27942 | 4210864 |
| Mb1714 | PRJNA682618 | SAMN17004158 | SRR13199753 | NA | Portugal | 2011 | C | Eu2 | 788996 | 165 | 58136 | 30419 | 58052 | 27089 | 4226766 |
| Mb1744 | PRJNA682618 | SAMN17004159 | SRR13199752 | NA | Portugal | 2012 | WB | w/o CC | 1796448 | 123 | 70851 | 44451 | 68962 | 37838 | 4240116 |
| Mb1746 | PRJNA682618 | SAMN17004160 | SRR13199751 | NA | Portugal | 2012 | RD | Eu2 | 1728552 | 116 | 83718 | 47787 | 73855 | 37225 | 4253747 |
| Mb1758 | PRJNA682618 | SAMN17004161 | SRR13199749 | NA | Portugal | 2012 | C | Eu2 | 1732898 | 113 | 83967 | 47786 | 80894 | 37191 | 4251022 |
| Mb1769 | PRJNA682618 | SAMN17004162 | SRR13199748 | NA | Portugal | 2012 | WB | Eu2 | 1374917 | 116 | 80074 | 48117 | 78283 | 35213 | 4182786 |
| Mb1785 | PRJNA682618 | SAMN17004163 | SRR13199747 | NA | Portugal | 2012 | RD | Eu2 | 940707 | 123 | 78244 | 37284 | 64130 | 34881 | 4241895 |
| Mb1789 | PRJNA682618 | SAMN17004164 | SRR13199746 | NA | Portugal | 2012 | C | Eu2 | 970309 | 163 | 57461 | 30334 | 53663 | 27184 | 4250449 |
| Mb1841 | PRJNA682618 | SAMN17004165 | SRR13199745 | NA | Portugal | 2012 | C | Eu2 | 1767230 | 126 | 79597 | 43101 | 79597 | 37228 | 4258855 |
| Mb1870 | PRJNA682618 | SAMN17004166 | SRR13199744 | NA | Portugal | 2012 | WB | Eu2 | 1395146 | 122 | 83370 | 42863 | 78910 | 37253 | 4270754 |
| Mb1915 | PRJNA682618 | SAMN17004167 | SRR13199743 | NA | Portugal | 2013 | RD | Eu2 | 1423501 | 117 | 80389 | 43805 | 78252 | 36107 | 4250774 |
| Mb1948 | PRJNA682618 | SAMN17004168 | SRR13199742 | NA | Portugal | 2013 | RD | w/o CC | 1523134 | 129 | 68000 | 39632 | 67967 | 36419 | 4263754 |
| Mb1960 | PRJNA682618 | SAMN17004169 | SRR13199741 | NA | Portugal | 2013 | RD | Eu2 | 1712114 | 117 | 80811 | 43643 | 78863 | 37191 | 4210417 |
| Mb2026 | PRJNA682618 | SAMN17004170 | SRR13199740 | NA | Portugal | 2013 | C | Eu2 | 1236634 | 128 | 70093 | 37125 | 68787 | 33783 | 4167064 |
| Mb2043 | PRJNA682618 | SAMN17004171 | SRR13199738 | NA | Portugal | 2013 | RD | Eu2 | 966319 | 129 | 60476 | 37125 | 60079 | 30506 | 4242591 |
| Mb2067 | PRJNA682618 | SAMN17004172 | SRR1319973 | NA | Portugal | 2013 | WB | Eu2 | 2004187 | 124 | 78197 | 42445 | 68641 | 39025 | 4266017 |
| Mb2206 | PRJNA682618 | SAMN17004173 | SRR13199736 | NA | Portugal | 2014 | C | Eu2 | 1751017 | 107 | 105527 | 47727 | 79003 | 40287 | 4220502 |
| Mb2235 | PRJNA682618 | SAMN17004174 | SRR13199735 | NA | Portugal | 2014 | RD | w/o CC | 1891547 | 121 | 75640 | 42823 | 73863 | 39470 | 4265718 |
| Mb2277 | PRJNA682618 | SAMN17004176 | SRR13199733 | NA | Portugal | 2014 | RD | w/o CC | 586402 | 151 | 59870 | 30428 | 50810 | 26162 | 4233355 |
| Mb2300 | PRJNA682618 | SAMN17004177 | SRR13199732 | NA | Portugal | 2014 | WB | Eu2 | 2833579 | 113 | 83265 | 43720 | 79050 | 40288 | 4265377 |
| Mb2310 | PRJNA682618 | SAMN17004178 | SRR13199731 | NA | Portugal | 2015 | RD | Eu2 | 1290912 | 127 | 73016 | 44682 | 72992 | 41752 | 4256966 |
| Mb2313 | PRJNA682618 | SAMN17004179 | SRR13199730 | NA | Portugal | 2015 | WB | Eu2 | 1671813 | 119 | 83205 | 40400 | 78317 | 36093 | 4261420 |
| Mb2325 | PRJNA682618 | SAMN17004180 | SRR13199729 | NA | Portugal | 2015 | RD | Eu2 | 1687836 | 115 | 81890 | 42759 | 75939 | 37125 | 4175664 |
| Mb2328 | PRJNA682618 | SAMN17004181 | SRR13199727 | NA | Portugal | 2015 | RD | Eu2 | 1220275 | 116 | 83842 | 46316 | 80665 | 39414 | 4254159 |
| Mb2347 | PRJNA682618 | SAMN17004182 | SRR13199726 | NA | Portugal | 2015 | WB | w/o CC | 1135051 | 122 | 66072 | 40212 | 63992 | 37123 | 4240191 |
| Mb2395 | PRJNA682618 | SAMN17004183 | SRR13199725 | NA | Portugal | 2015 | WB | Eu2 | 2365469 | 114 | 80423 | 43380 | 79050 | 37219 | 4265753 |
| Mb2397 | PRJNA682618 | SAMN17004184 | SRR13199724 | NA | Portugal | 2015 | WB | Eu2 | 2171022 | 113 | 83584 | 48121 | 78324 | 43376 | 4261534 |
| Mb502499 | PRJEB4884 | SAMEA2340906 | SRR502499 | NA | Ghana | NA | H | Af1 | 27469960 | 128 | 98630 | 60477 | 96086 | 53360 | 4289661 |
| Mb502526 | PRJEB4884 | SAMEA2340934 | SRR502526 | NA | Ghana | NA | H | Af1 | 35597804 | 131 | 96092 | 60395 | 92404 | 52815 | 4289813 |
| Mb1203064 | PRJEB4884 | SAMEA3504611 | SRR1203064 | NA | Ghana | NA | H | Af1 | 4093722 | 119 | 96134 | 62878 | 89837 | 53934 | 4283215 |
| Mb4117155 | PRJNA341471 | SAMN05725038 | SRR4117155 | NA | France | NA | WB | Af2 | 1,929,897 | 99 | 80623 | 48024 | 68832 | 37868 | 4244731 |
| Mb1791710 | PRJNA251692 | SAMN03300068 | SRR1791710 | NA | Tanzania | NA | Ch | Af2 | 2120788 | 161 | 64008 | 33852 | 57230 | 30067 | 4242025 |
| Mb1791712 | PRJNA251692 | SAMN03300070 | SRR1791712 | NA | Tanzania | NA | Ch | Af2 | 1226608 | 165 | 64008 | 32362 | 58095 | 29987 | 4229041 |
| Mb1792006 | PRJNA251692 | SAMN03300364 | SRR1792006 | NA | USA | 2006 | C | Eu1 | 2929588 | 148 | 71509 | 35833 | 63983 | 30811 | 4242682 |
| Mb1792127 | PRJNA251692 | SAMN03300485 | SRR1792127 | NA | USA | 2008 | C | Eu1 | 2604288 | 166 | 63983 | 31064 | 53658 | 29655 | 4239161 |
| Mb1792361 | PRJNA251692 | SAMN03300719 | SRR1792361 | NA | USA | 2013 | C | Eu1 | 1652284 | 159 | 58037 | 31835 | 53668 | 30110 | 4251183 |
| Mb7240242 | PRJNA251692 | SAMN09284284 | SRR7240242 | NA | USA | 2016 | C | Eu1 | 1410214 | 168 | 53817 | 30375 | 53663 | 27094 | 4225345 |
| Mb7240415 | PRJNA251692 | SAMN09284172 | SRR7240415 | NA | USA | 2014 | C | Eu1 | 1632424 | 162 | 55811 | 30088 | 52223 | 26456 | 4235702 |
| Mb1791984 | PRJNA251692 | SAMN03300342 | SRR1791984 | NA | USA | 2005 | C | Eu1 | 1632424 | 153 | 60474 | 34666 | 58036 | 31333 | 4,255,572 |
| MBE1 | PRJNA471317 | SAMN09206576 | NA | NZ_QFZD00000000.1 | Egypt | 2014 | C | w/o CC | NA | 8 | 2859506 | 567488 | 2859190 | 429315 | 4348120 |
| MBE3 | PRJNA471317 | SAMN09206578 | NA | NZ_QFZB00000000.1 | Egypt | 2014 | C | w/o CC | NA | 8 | 2858537 | 567224 | 2858221 | 428747 | 4346664 |
| MBE4 | PRJNA471317 | SAMN09206579 | NA | NZ_QFZA00000000.1 | Egypt | 2014 | C | w/oCC | NA | 8 | 3176044 | 940186 | 3175483 | 801561 | 4347513 |
| MBE10 | PRJNA471317 | SAMN09206584 | NA | NZ_QFYV00000000.1 | Egypt | 2015 | C | w/o CC | NA | 10 | 3667979 | 3667979 | 2772194 | 891590 | 4351970 |
| Mb0077 | PRJNA428763 | SAMN08321035 | NA | NZ_PUEG00000000.1 | Canada | 2006 | E | w/o CC | NA | 3 | 4328040 | 4328040 | 2242838 | 2083316 | 4343450 |
| Mb0565 | PRJNA428763 | SAMN08321046 | SRR6467885 | NZ_PUFV00000000.1 | Canada | 2011 | C | w/o CC | NA | 2 | 4334518 | 4334518 | 334835 | 243038 | 4338276 |
| BMR25 | PRJNA428763 | SAMN08321055 | SRR6467887 | NZ_PUEH01000003.1 | Canada | 1985 | B | w/o CC | NA | 7 | 1338840 | 1338840 | 600419 | 515461 | 4327606 |
| Mb3601 | PRJEB33636 | SAMEA5803801 | SRR6467891 | NZ_LR699570.1 | France | 2014 | C | Eu3 | NA | 1 | NA | NA | NA | NA | 4365068 |
| Mb0476 | PRJNA428763 | SAMN08321034 | SRR6467889 | NZ_CP027035.1 | Canada | 2002 | C | Eu2 | NA | 1 | NA | NA | NA | NA | 4355637 |
| MbSP38 | PRJNA273066 | SAMN03288261 | SRR6705904 | NZ_CP015773.2 | Brazil | 2010 | C | Eu2 | NA | 1 | NA | NA | NA | NA | 4347648 |
| Mb1595 | PRJNA285833 | SAMN03761171 | NA | NZ_CP012095.1 | Korea | 2012 | C | w/o CC | NA | 1 | NA | NA | NA | NA | 4351712 |
| Mb0030 | PRJNA270004 | SAMN03257089 | NA | CP010332.1 | China | NA | NA | w/o CC | NA | 1 | NA | NA | NA | NA | 4336227 |
| Mb0001 | PRJNA546139 | SAMN11954087 | NA | NZ_CP040832.1 | Brazil | 2015 | TP | Eu2 | NA | 1 | NA | NA | NA | NA | 4349904 |
| Mb0003 | PRJNA412319 | SAMN07709478 | NA | NZ_CP023708.2 | India | 1986 | C | w/o CC | NA | 1 | NA | NA | NA | NA | 4350102 |
| Mb31150 | PRJNA233393 | SAMN02567761 | SRR1173570 | NZ_JKAM00000000.1 | Uganda | NA | Ch | Af2 | NA | 1 | NA | NA | NA | NA | 4272013 |
| MbAF2122/97 | PRJEB15187 | SAMEA20450668 | ERS1465382 | LT708304.1 | United Kingdom | 1997 | C | Eu1 | NA | 1 | NA | NA | NA | NA | 4272013 |

**Supplementary Table 1 (cont.)**

| ***M.* *bovis* ID** | **Genome fraction ^(d)^** | **Largest contig (bp) ^(c)^** | **%GC ^(d)^** | **Fully unassemble contigs ^(d)^** | **Genome coverage ^(e)^** | **Average coverage ^(e)^** |
| --- | --- | --- | --- | --- | --- | --- |
| Mb0220 | 96,98 | 227395 | 65,5 | 0 | 99.69 | 126.7 |
| Mb0261 | 99,116 | 352999 | 65,6 | 0 | 99.92 | 368.8 |
| Mb0601 | 97,768 | 227395 | 65,5 | 0 | 99.73 | 98.7 |
| Mb0769 | 97,981 | 227396 | 65,5 | 0 | 99.68 | 108.1 |
| Mb0783 | 97,781 | 227394 | 65,5 | 0 | 99.66 | 105.5 |
| Mb0865 | 96,886 | 260130 | 65,5 | 0 | 98.79 | 73.7 |
| Mb0891 | 99,133 | 296081 | 65,6 | 0 | 99.91 | 345.4 |
| Mb0893 | 97,159 | 236826 | 65,5 | 0 | 99.66 | 100.6 |
| Mb1317 | 97,911 | 227395 | 65,5 | 0 | 99.59 | 91.3 |
| Mb1339 | 97,954 | 227394 | 65,5 | 0 | 99.63 | 87.2 |
| Mb1458 | 97,956 | 227395 | 65,5 | 0 | 99.67 | 103.1 |
| Mb1480 | 96,374 | 227395 | 65,5 | 0 | 99.64 | 109.7 |
| Mb1654 | 96,292 | 182443 | 65,4 | 0 | 99.34 | 33.3 |
| Mb1670 | 97,857 | 227395 | 65,5 | 0 | 99.64 | 110.1 |
| Mb1711 | 97,661 | 236608 | 65,4 | 0 | 99.64 | 93.2 |
| Mb1712 | 96,701 | 227395 | 65,4 | 0 | 99.05 | 55.6 |
| Mb1714 | 97,049 | 182443 | 65,4 | 0 | 99.37 | 40.5 |
| Mb1744 | 97,326 | 227395 | 65,5 | 0 | 99.69 | 92.5 |
| Mb1746 | 97,67 | 227398 | 65,5 | 0 | 99.7 | 88.1 |
| Mb1758 | 97,585 | 227395 | 65,5 | 0 | 99.52 | 93.4 |
| Mb1769 | 96,021 | 227395 | 65,5 | 0 | 99.15 | 72 |
| Mb1785 | 97,43 | 227392 | 65,4 | 0 | 99.56 | 49.5 |
| Mb1789 | 97,57 | 175580 | 65,5 | 0 | 99.49 | 50.8 |
| Mb1841 | 97,791 | 227394 | 65,5 | 0 | 99.67 | 91.2 |
| Mb1870 | 98,091 | 236657 | 65,5 | 0 | 99.64 | 67.7 |
| Mb1915 | 97,612 | 227394 | 65,4 | 0 | 99.65 | 74 |
| Mb1948 | 97,861 | 227395 | 65,5 | 0 | 99.66 | 79.4 |
| Mb1960 | 96,702 | 242209 | 65,5 | 0 | 99.55 | 91.3 |
| Mb2026 | 95,679 | 227394 | 65,4 | 0 | 99.61 | 64.2 |
| Mb2043 | 97,416 | 227395 | 65,4 | 0 | 99.41 | 50.3 |
| Mb2067 | 97,912 | 227392 | 65,5 | 0 | 99.71 | 105.1 |
| Mb2206 | 96,922 | 353687 | 65,5 | 0 | 99.72 | 92.7 |
| Mb2235 | 97,933 | 227395 | 65,5 | 0 | 99.65 | 100.4 |
| Mb2277 | 96,966 | 225249 | 65,4 | 0 | 99.36 | 29.8 |
| Mb2300 | 97,912 | 227398 | 65,5 | 0 | 99.75 | 149.2 |
| Mb2310 | 97,739 | 227394 | 65,5 | 0 | 99.63 | 65.8 |
| Mb2313 | 97,815 | 227410 | 65,5 | 0 | 99.65 | 85.3 |
| Mb2325 | 95,873 | 227395 | 65,5 | 0 | 99.67 | 86.7 |
| Mb2328 | 97,653 | 227394 | 65,5 | 0 | 99.48 | 65.4 |
| Mb2347 | 97,333 | 227395 | 65,4 | 0 | 99.56 | 60.4 |
| Mb2395 | 97,954 | 227398 | 65,5 | 0 | 99.74 | 124.4 |
| Mb2397 | 97,825 | 227398 | 65,5 | 0 | 99.58 | 116.4 |
| Mb502499 | 98,533 | 229023 | 65,6 | 0 | NA | NA |
| Mb502526 | 98,568 | 229031 | 65,6 | 0 | NA | NA |
| Mb1203064 | 98,397 | 266790 | 65,6 | 0 | NA | NA |
| Mb4117155 | 97,476 | 161970 | 65,5 | 0 | NA | NA |
| Mb1791710 | 97,379 | 209663 | 65,5 | 0 | NA | NA |
| Mb1791712 | 97,006 | 181601 | 65,5 | 0 | NA | NA |
| Mb1792006 | 97,467 | 182360 | 65,5 | 0 | NA | NA |
| Mb1792127 | 97,471 | 182249 | 65,6 | 0 | NA | NA |
| Mb1792361 | 97,725 | 182360 | 65,6 | 0 | NA | NA |
| Mb7240242 | 97,096 | 182444 | 65,4 | 0 | NA | NA |
| Mb7240415 | 97,367 | 182361 | 65,4 | 0 | NA | NA |
| Mb1791984 | 97,838 | 206336 | 65,5 | 0 | NA | NA |
| MBE1 | 99,893 | 2859506 | 65,6 | 0 | NA | NA |
| MBE3 | 99,883 | 2858537 | 65,6 | 0 | NA | NA |
| MBE4 | 99,872 | 3176044 | 65,6 | 0 | NA | NA |
| MBE10 | 99,933 | 3667979 | 65,6 | 0 | NA | NA |
| Mb0077 | 99,842 | 4328040 | 65,6 | 0 | NA | NA |
| Mb0565 | 99,081 | 4334518 | 65,6 | 0 | NA | NA |
| BMR25 | 99,271 | 2026723 | 65,6 | 0 | NA | NA |
| Mb3601 | NA | NA | 65,6 | NA | NA | NA |
| Mb0476 | NA | NA | 65,6 | NA | NA | NA |
| MbSP38 | NA | NA | 65,6 | NA | NA | NA |
| Mb1595 | NA | NA | 65,6 | NA | NA | NA |
| Mb0030 | NA | NA | 65,6 | NA | NA | NA |
| Mb0001 | NA | NA | 65,6 | NA | NA | NA |
| Mb0003 | NA | NA | 65,6 | NA | NA | NA |
| Mb31150 | NA | NA | 65,5 | NA | NA | NA |
| MbAF2122/97 | NA | NA | 65,5 | NA | NA | NA |

(a) Host species: C: cattle; Ch: chimpanzee; E: elk; H: human; RD: red deer; TP: Tapirus terrestris; WB: wild boar

(b) CC: Clonal complex; Eu1: European 1, Eu2: European 2, Eu3: European 3, Af1: African 1, Af2: African 2, and w CC: without clonal complex

(c) Parameters obtained after *de novo* genome assembly performed by Unicycler pipeline.

(d) Parameters obtained by QUAST pipeline.

(e) Parameters obtained by vSNP pipeline.

NA: non-available information/non-aplicable.

**Supplementary Table 2.** Genes included the HGT group. List of genes, putative function and synonymous and non-synonymous SNPs.

| **Gene name** | **Mb** | **Function** | **Total SNPs in this dataset** | **Syn SNPs** | **NS SNPs** |
| --- | --- | --- | --- | --- | --- |
| *acpA* | Mb0034 | Probable acyl carrier protein acpA | - |  |  |
| *amiD* | Mb3409 | Probable amidase amiD | - |  |  |
| *atsD* | Mb0682 | Possible arylsulfatase atsD | 4 | 1 | 3 |
| *bioF2* | Mb0033 | Possible 8-amino-7-oxononanoate synthase BioF2 | 1 | 1 |  |
| *cdh* | Mb2311 | Probable CDP-diacylglycerol pyrophosphatase cdh | 1 |  | 1 |
| *celA2a* | Mb1119 | Probable cellulase celA2a | - |  |  |
| *celA2b* | Mb1120 | Probable cellulase celA2b | - |  |  |
| *cyp128* | Mb2291c | Probable cytochrome P450, cyp128 | - |  |  |
| *cyp135A1* | Mb0334c | Possible cytochrome P450, cyp135A1 | - |  |  |
| *cyp141* | Mb3144 | Probable cytochrome P450, cyp141 | - |  |  |
| *echA18* | Mb3408 | Probable enoyl-coa hydratase (fragment) echa18.1 | - |  |  |
| *erm(37)* | Mb2010 | Probable 23s rrna methyltransferase erm(37) | 1 |  | 1 |
| *frdA* | Mb1578 | Probable fumarate reductase frdA | 1 |  | 1 |
| *frdB* | Mb1579 | Probable fumarate reductase frdB | - |  |  |
| *frdC* | Mb1579 | Probable fumarate reductase frdC | - |  |  |
| *frdD* | Mb1580 | Probable fumarate reductase frdD | - |  |  |
| *gca* | Mb0116 | GDP-mannose-4,6-dehydratase | 1 |  | 1 |
| *gmhA* | Mb0117 | Probable sedoheptulose-7-phosphate isomerase gmhA | - |  |  |
| *gmhB* | Mb0118 | Possible d-alpha,beta-d-heptose-1,7-biphosphate phosphatase gmhB | 1 |  | 1 |
| *grcC2* | Mb1016c | Probable polyprenyl-diphosphase synthase grcC2 | - |  |  |
| *greA* | Mb1109c | Transcription elongation greA | - |  |  |
| *hddA* | Mb0119 | Possible d-alpha-d-heptose-7-phosphate kinase hddA | 2 | 1 | 1 |
| *hsdS* | Mb2782c | Possible type I restriction/modification system specificity determinant hsdS | 1 |  | 1 |
| *hycD* | Mb0087 | Probable formate hydrogenlyase hycD | 1 | 1 |  |
| *hycE* | Mb0090 | Possible formate hydrogenase hycE | 2 |  | 2 |
| *hycP* | Mb0088 | Possible hydrogenase hycP | - |  |  |
| *hycQ* | Mb0089 | Possible hydrogenase hycQ | - |  |  |
| *lppG* | Mb1981c | Possible conserved lipoprotein lppG | 1 |  | 1 |
| *lppOa* | Mb2312 | Probable conserved lipoprotein lppOa | - |  |  |
| *lppOb* | Mb2313 | Probable conserved lipoprotein lppOb | 2 | 1 | 1 |
| *lpqO* | Mb0620 | Probable conserved lipoprotein lpqO | - |  |  |
| *lprI* | Mb1568c | Possible lipoprotein lprL | - |  |  |
| *mazE2* | Mb0679c | Possible antitox mazE2 | - |  |  |
| *mazF2* | Mb0678c | Toxin mazf2 | - |  |  |
| *mazF6* | Mb2014c | Toxin mazf6 | - |  |  |
| *mazF9* | Mb2824c | Toxin MazF9 | - |  |  |
| *mbtL* | Mb1379 | Acyl carrier protein | - |  |  |
| *mesTa* | Mb3202c | Probable epoxide hydrolase mesTa | - |  |  |
| *mesTb* | Mb3201c | Probable epoxide hydrolase mesTb | 1 |  | 1 |
| *mmpL9a* | Mb2367 | Probable conserved transmembrane transport protein mmpL9a | - |  |  |
| *mmpL9b* | Mb2368 | Probable conserved transmembrane transport protein mmpL9b | - |  |  |
| *moaA1* | Mb3136 | Molybdenum cofactor biosynthesis protein A | - |  |  |
| *moaB1* | Mb3137 | Probable pterin-4-alpha-carbinolamine dehydratase moaB1 | - |  |  |
| *moaC1* | Mb3138 | Molybdenum cofactor biosynthesis protein C | - |  |  |
| *moaD1* | Mb3139 | Molybdenum cofactor biosynthesis protein D | - |  |  |
| *moaR1* | Mb3147 | Probable transcriptional regulatory protein | - |  |  |
| *moeB2* | Mb3143 | Probable pterin-4-alpha-carbinolamine dehydratase moaB2 | - |  |  |
| *moeW* | Mb2366c | Molybdopterin biosynthesis protein W | 1 |  | 1 |
| *nadR* | Mb0218c | Possible transcriptional regulatory protein nadR | - |  |  |
| *OmpA* | Mb0923 | Outer membrane protein A, ompA | 1 |  | 1 |
| *pflA* | Mb3162 | Pyruvate formate lyase activating enzyme | 1 | 1 |  |
| *pknJ* | Mb2115 | Transmembrane serine/threonine-protein kinase J | 1 |  | 1 |
| *SecE2* | Mb0386 | Possible protein transport protein SecE2 | - |  |  |
| *tcrA* | Mb0618c | Probable two-component DNA-binding response regulator tcrA | - |  |  |
| *vapB10* | Mb1433c | Possible antitoxin VapB10 | - |  |  |
| *VapB2* | Mb0308 | Possible toxic vapb2 | - |  |  |
| *vapB27* | Mb0615c | Possible toxin vapB27 | - |  |  |
| *vapB28* | Mb0624 | Possible antitoxin vapb28 | - |  |  |
| *vapB31* | Mb0769 | Possible antitoxin VapB31 | - |  |  |
| *vapB38* | Mb2521 | Possible antitoxin vapb38 | - |  |  |
| *vapB4* | Mb0612c | Possible antitoxin vapb4 | - |  |  |
| *vapB43* | Mb2896 | Possible antitoxin VapB43 | - |  |  |
| *vapB6* | Mb0676c | Possible antitoxin vapb6 | - |  |  |
| *vapB7* | Mb0681c | Possible antitoxin vapb7 | - |  |  |
| *vapB8* | Mb0683 | Possible antitoxin vapb8 | - |  |  |
| *vapC10* | Mb1432c | Possible toxin VapC10 | - |  |  |
| *VapC2* | Mb0309 | Possible toxic vapc2 | 1 |  | 1 |
| *vapC27* | Mb0614c | Possible toxin vapc27 | - |  |  |
| *vapC28* | Mb0625 | Possible toxin vapc28 | - |  |  |
| *vapC31* | Mb0770 | Possible toxin vapc31 | 1 |  | 1 |
| *vapC38* | Mb2522 | Possible toxin VapC38 | - |  |  |
| *vapC4* | Mb0611c | Possible toxin vapc4 | - |  |  |
| *vapC43* | Mb2897 | Possible toxin vapc43 | - |  |  |
| *vapC6* | Mb0675c | Possible toxin vapC6 | - |  |  |
| *vapC7* | Mb0680c | Possible toxin vapc7 | - |  |  |
| *vapC8* | Mb0684 | Possible toxin vapc8 | - |  |  |
| *wbbL2* | Mb1552 | Possible rhamnosyl transferase wbbL2 | - |  |  |

Legend: Syn SNPs: synonymous SNPs; NS SNPs: non-synonymous SNPs.

**Supplementary Table 3.** Genes included the 3R group. List of genes, putative function and synonymous and non-synonymous SNPs.

| **Gene name** | **Mb** | **Function** | **Total SNPs in this dataset** | **Syn SNPs** | **NS SNPs** |
| --- | --- | --- | --- | --- | --- |
| *alkAa* | Mb1351c | Probable ada regulatory protein alkAa | - |  |  |
| *alkAb* | Mb1350c | Probable ada regulatory protein alkAb | - |  |  |
| *deoA* | Mb3343c | Probable thymidine phosphohydrolase | - |  |  |
| *dinF* | Mb2861c | Possible DNA-damage-inducible protein F | - |  |  |
| *dinP* | Mb3082 | Possible DNA-damageinducible protein P | - |  |  |
| *dinx* | Mb1564 | Probable DNA polymerase IV | 1 |  | 1 |
| *dnaN* | Mb0002 | Probable DNA polymerase III | - |  |  |
| *dnaQ* | Mb3738c | Probable DNA polymerase III | - |  |  |
| *dnaZX* | Mb3748c | DNA polymerase III | 1 | 1 |  |
| *dut* | Mb2716c | Probable dUTPase | - |  |  |
| *end* | Mb0689 | Probable endonuclease IV | - |  |  |
| *lexA* | Mb2739 | Repressor lexA | 1 |  | 1 |
| *ligA* | Mb3039c | Probable DNA ligase | - |  |  |
| *ligB* | Mb3089 | Possible ATP-dependent ligase ligB | 1 | 1 |  |
| *ligC* | Mb3758 | Possible ATP-dependent ligase ligC | - |  |  |
| *ligD* | Mb0963 | Possible ATP-dependent ligase ligD | 1 |  | 1 |
| *mfd* | Mb1048 | Probable transcription repair coupling factor | 3 | 2 | 1 |
| *mpg* | Mb1714 | Possible 3-methyladenine DNA glycosylase | 1 |  | 1 |
| *mrr* | Mb2557c | Probable restriction system protein | 1 |  | 1 |
| *mutT1* | Mb3009 | Possible hydrolase | - |  |  |
| *mutT2* | Mb1192 | Probable mutator protein | 2 |  | 2 |
| *mutT3* | Mb0421 | Probable mutator protein | - |  |  |
| *mutT4* | Mb3938 | Probable mutator protein | - |  |  |
| *mutY* | Mb3620 | Probable adenine glycosylase | - |  |  |
| *nei* | Mb3325 | Probable endonuclease VIII | 1 |  | 1 |
| *nth* | Mb3698c | Probable endonuclease III | 1 |  | 1 |
| *nudC* | Mb3224c | Probable NADH pyrophosphatase | 2 | 1 | 1 |
| *ogt* | Mb1349c | 6-O-methylguanine-DNA methyltransferase | - |  |  |
| *polA* | Mb1655 | Probable DNA polymerase I | 1 | 1 |  |
| *radA* | Mb3616 | DNA repair protein | 1 | 1 |  |
| *recA* | Mb2756c | Recombinase A | 1 | 1 |  |
| *recBa* | Mb0647c | Probable exonuclease V, recBa | 1 |  | 1 |
| *recBb* | Mb0646c | Probable exonuclease V, recBb | - |  |  |
| *recC* | Mb0648c | Probable exonuclease V, recC | 1 |  | 1 |
| recD | Mb0645c | Probable exonuclease V | - |  |  |
| *recF* | Mb0003 | DNA replication and repair protein | - |  |  |
| *recG* | Mb2998c | Possible ATP-dependent DNA helicase | 2 |  | 2 |
| *recN* | Mb1722 | Probable DNA repair protein | 1 |  | 1 |
| *recO* | Mb2383c | Possible DNA repair protein | 1 | 1 |  |
| *recR* | Mb3742c | Probable recombination protein | - |  |  |
| *recX* | Mb2755c | Regulatory protein | - |  |  |
| *ruvA* | Mb2624c | Probable holiday junction DNA helicase | - |  |  |
| *ruvB* | Mb2623c | Probable holiday junction DNA helicase | - |  |  |
| *ruvC* | Mb2625c | Probable crossover junction endodeoxyribonuclease | - |  |  |
| *ssb* | Mb0055 | Probable single-strand binding protein | - |  |  |
| *tagA* | Mb1242 | Probable DNA-3-methyladenine glycosylase I | - |  |  |
| *ung* | Mb3000c | Probable uracil-DNA glycosylase | - |  |  |
| *uvrA* | Mb1664 | Probable excinuclease ABC | 1 |  | 1 |
| *uvrB* | Mb1659 | Probable excinuclease ABC | - |  |  |
| *uvrC* | Mb1455 | Probable excinuclease ABC | - |  |  |
| *uvrD1* | Mb0974 | Possible ATP-dependent DNA helicase II | - |  |  |
| *uvrD2* | Mb3222c | Possible ATP-dependent DNA helicase II | - |  |  |
| *xthA* | Mb0435c | Probable exodeoxyribonuclease III | - |  |  |

Legend: Syn SNPs: synonymous SNPs; NS SNPs: non-synonymous SNPs.


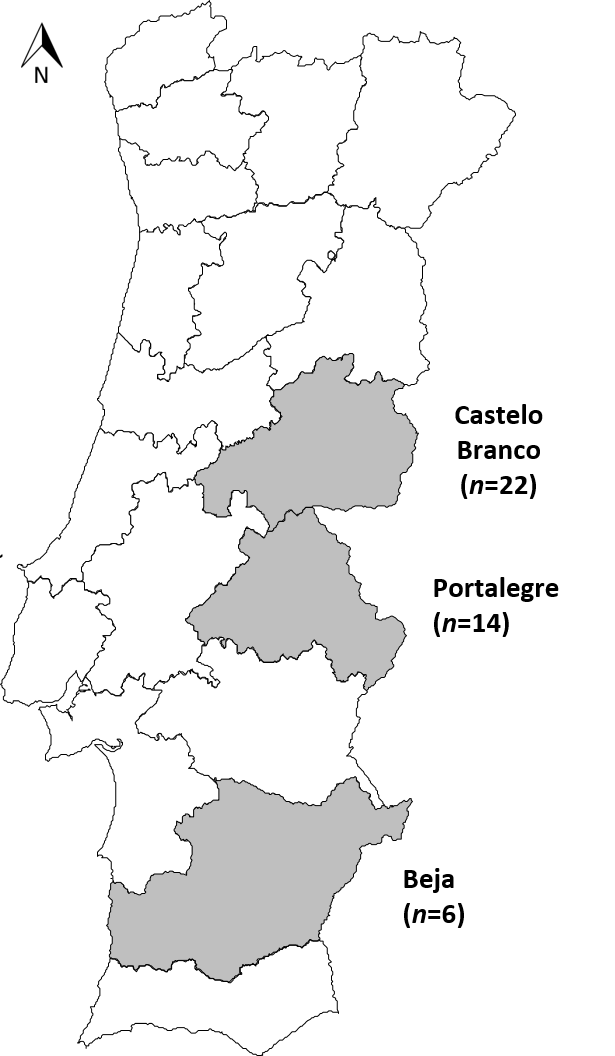


**Supplementary Figure 1**. Geographic location, represented at the district (administrative level sample unit), of the 42 newly sequenced *M. bovis* isolates from Portugal. Map was generated with QGIS software (version 3.10, https ://www.qgis.org/en/site/).


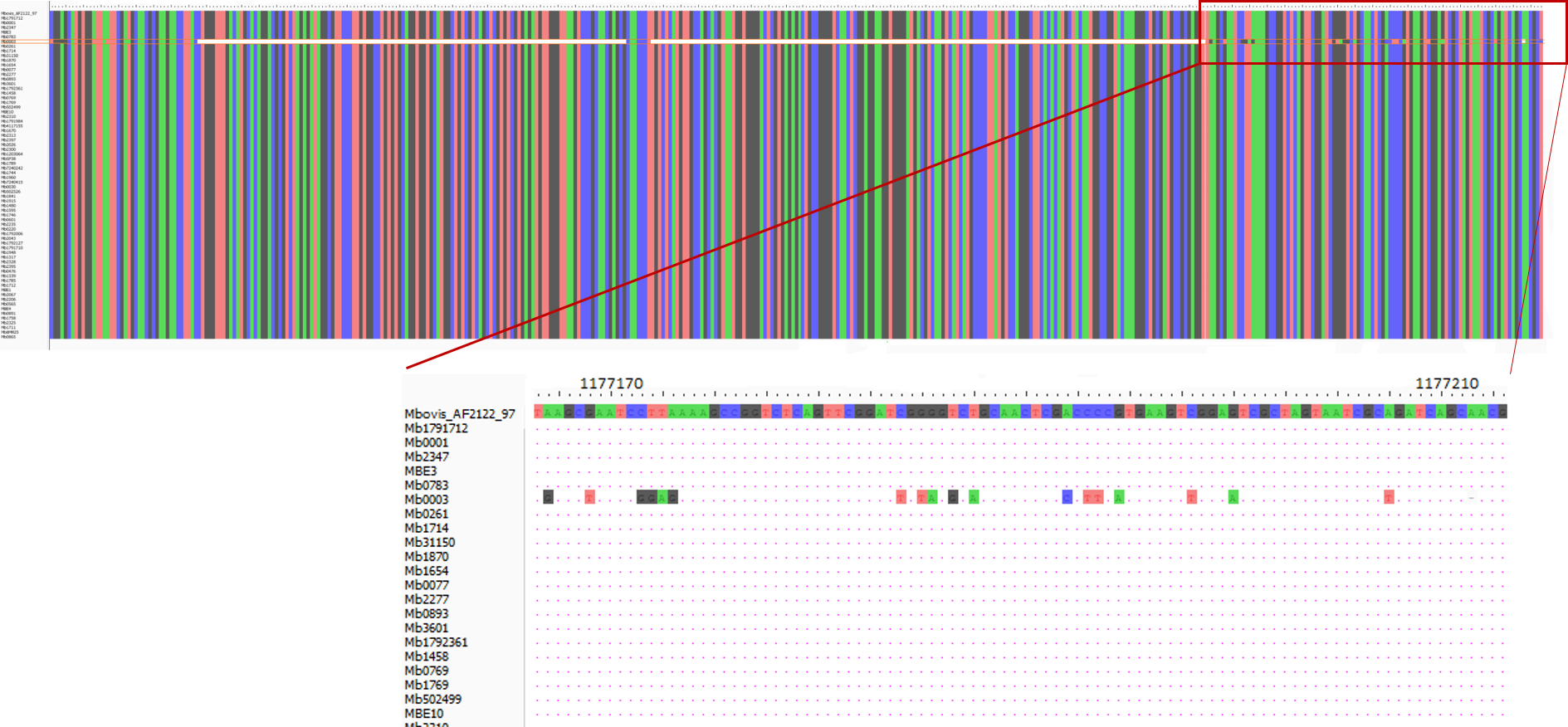


**Supplementary Figure 2.** Detailed visualization of alignment in recombination region of *M. bovis* dataset affecting the *rrs* gene.


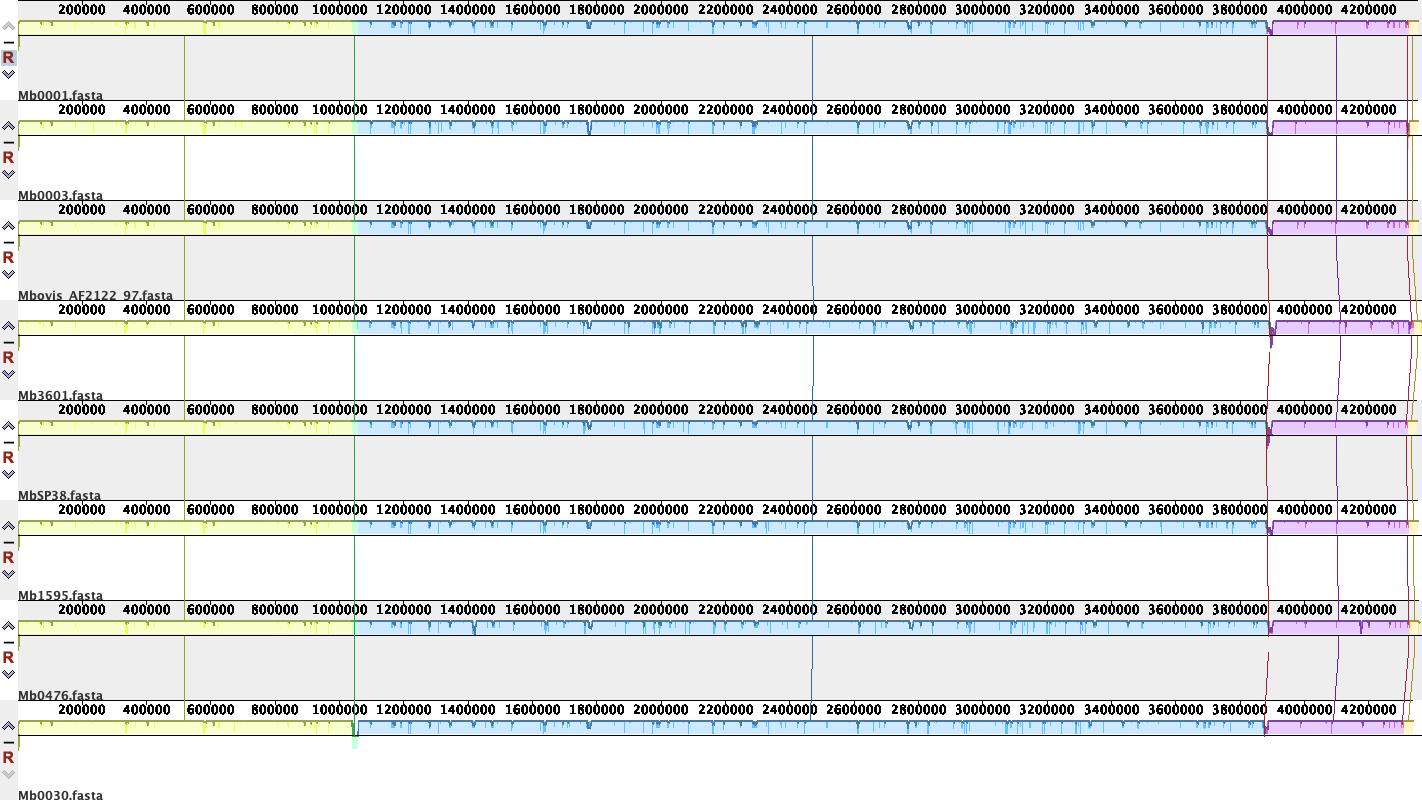


**Supplementary Figure 3.** Multi-genome alignment of complete genomes performed with MAUVE. The colinear blocks are identified by different colors.


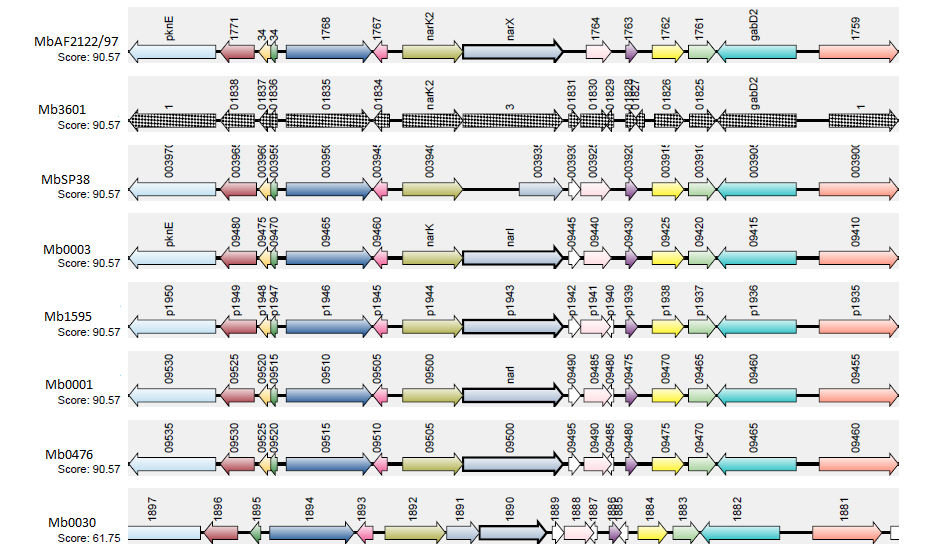


**Supplementary Figure 4.** Synteny scores for *narX* as predicted by SyntTax.


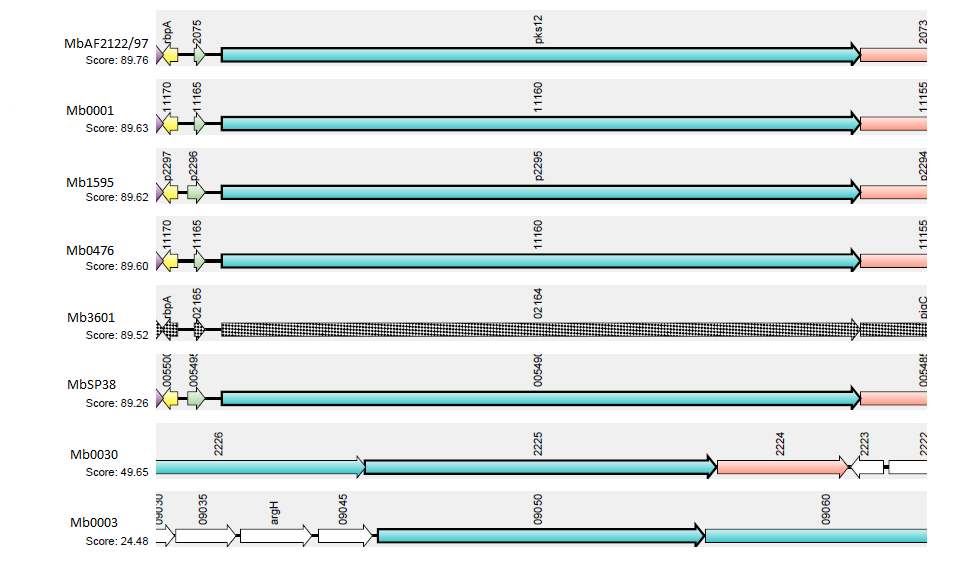


**Supplementary Figure 5.** Synteny scores for *pks12* as predicted by SyntTax.
